# Identifying the sources of structural sensitivity in partially specified biological models

**DOI:** 10.1038/s41598-020-73710-z

**Published:** 2020-10-09

**Authors:** Matthew W. Adamson, Andrew Yu. Morozov

**Affiliations:** 1 Institute of Mathematics, Institute of Environmental Systems Research, University of Osnabrück, Osnabrück, 49076 Germany; 2 Department of Mathematics, University of Leicester, Leicester, LE1 7RH UK; 3 Institute of Ecology and Evolution, Russian Academy of Sciences, 33 Leninskii pr., Moscow, Russia 119071; 4 N.I. Lobachevsky State University of Nizhny Novgorod, Nizhny Novgorod, Russia

**Keywords:** Biological models, Ecological modelling, Applied mathematics

## Abstract

Biological systems are characterised by a high degree of uncertainty and complexity, which implies that exact mathematical equations to describe biological processes cannot generally be justified. Moreover, models can exhibit sensitivity to the precise formulations of their component functions—a property known as structural sensitivity. Structural sensitivity can be revealed and quantified by considering partially specified models with uncertain functions, but this goes beyond well-established, parameter-based sensitivity analysis, and currently presents a mathematical challenge. Here we build upon previous work in this direction by addressing the crucial question of identifying the processes which act as the major sources of model uncertainty and those which are less influential. To achieve this goal, we introduce two related concepts: (1) the gradient of structural sensitivity, accounting for errors made in specifying unknown functions, and (2) the partial degree of sensitivity with respect to each function, a global measure of the uncertainty due to possible variation of the given function while the others are kept fixed. We propose an iterative framework of experiments and analysis to inform a heuristic reduction of structural sensitivity in a model. To demonstrate the framework introduced, we investigate the sources of structural sensitivity in a tritrophic food chain model.

## Introduction

Compared to the physical sciences, biological processes are often more complex, more variable and less well understood. Consequently, the uncertainty in any representation of biological processes by a simple ‘macroscale’ mathematical model applies to the choice of equations themselves, as well as their parameters. This can be problematic since models can be sensitive to the precise functions used to represent processes such as growth rates, mortality rates or feeding rates of organisms, a property known as structural sensitivity^1–3^, even when they are robust to variations of parameters for a given choice of functions. Since many if not all of the precise functions used in a biological model have little formal justification and may be considered to be uncertain, structural sensitivity is a challenge to quantifying model uncertainty and the sensitivity of the resulting predictions that must be addressed. In previous work, a novel framework was proposed to detect and quantify structural sensitivity in biological models when the exact formulation of a single model function is uncertain^3–5^. However, a fundamental open question concerns how to evaluate the respective contributions of each individual unspecified model function to the overall sensitivity of the model outputs. In this paper we demonstrate how this can be done through an extension of the authors’ previously developed framework to consider structural sensitivity with respect to each unknown model process.

Overall, the fact that the equations used to model many biological processes are not themselves justifiable is rarely appreciated in the literature (with some exceptions^2,6^). However, even the simplest biological processes involve dozens of complex interactions among diverse entities (proteins, cells, organs) across many different scales of time and space and levels of organisation, and can at best be approximated by a given function. Even if processes can be represented by mechanistically-derived functions as in the case of the Holling-type functional responses in predator-prey systems^7^, the derivation is often based upon a number of idealized assumptions and the resulting function is only justified when these assumptions approximately hold, and may not remain valid under the effects of evolution^8^, scaling up in space^9^ or changes in environmental conditions^10^. Most of the questions related to uncertainty in models are addressed either to the particular choice of model parameters^11^, or to the topology of the system coupling^12^—i.e. as represented in the flow diagram—while the actual equations used to represent individual processes are treated as fixed ‘modules’ which are plugged into models without their validity being questioned. Such an approach would be reasonable if close model functions could be guaranteed to produce consistent model predictions, but due to structural sensitivity this is not the case^3,13^.

There is a long tradition in mathematical biology of using general classes of models and proving results based on generic properties of the model functions^14,15^, stretching back to the work of Kolmogorov on predator-prey systems^16^. A more applied approach is the consideration of partially specified models^17,18^, whereby known functions are given a precise formulation, while unknown functions are represented by a set of biological constraints and either an objective functional measuring goodness of fit to data and penalising high curvature, or a set of error bounds together with bounds on the second derivatives. Other promising approaches are generalized modelling^6,19,20^, nonlinear forecasting approaches^21^, and the formation of a new model out of the parameterisation of the convex hull of several choices of model functions, followed by a standard bifurcation analysis on the combined model^22^. When the model functions themselves are not fixed, rigorously detecting and quantifying structural sensitivity in the system is challenging since the space of inputs that needs to be sampled from is infinite dimensional. An approach to do this with respect to the stability of an equilibrium was previously developed^3,4^ using partially specified models^17^. Unspecified functions are restricted to a certain neighbourhood which can be projected into the space of the local function values that influence the system’s Jacobian matrix, which is finite dimensional and can therefore be investigated directly. Quantifying the overall sensitivity of a system with respect to all of its uncertain inputs in this way is a task often called uncertainty analysis (UA). When the uncertain inputs are parameters, a large range of techniques for UA exists^23^.

A related task to uncertainty analysis is sensitivity analysis (SA). While UA determines the overall sensitivity of the model outputs given uncertain inputs, the aim of SA is to quantify the respective contribution of the different model inputs to this uncertainty. Such an analysis can have many uses. It can tell us which factors are most responsible for the uncertainty in a system, so that these can then be targeted for further investigation in order to reduce the uncertainty most effectively^23^. Conversely, SA can indicate factors to which a model is not sensitive and which can therefore be safely fixed as a model reduction strategy. SA can also indicate conceptual problems with a model and inform theory: highly sensitive model inputs can become targets for further refinement of the model formulation, for a re-evaluation of their role in the corresponding theory or for nonparametric analysis^24^. For example, the functional response of a highly sensitive predator prey model has previously been refined using the parameter cascades method^25^. As with UA, SA can be either local or global, with local SA being far more prevalent in the literature, even though it is only reliable when applied to linear models^26^. A number of further techniques apply local sensitivity analysis in a global way to obtain extra information or computational benefits^27,28^. Parameter-based techniques for SA are widespread, but when the model functions themselves are uncertain, techniques to carry out SA are lacking. The presence of structural sensitivity in models thus clearly raises the question of how we can extend sensitivity analysis to incorporate variation in the model functions.

In this paper we address sensitivity analysis with respect to multiple uncertain model functions. We propose a method to evaluate which uncertain processes contribute the most to the uncertainty in the model outputs and therefore would be the most useful to investigate in order to reduce the total structural sensitivity of the model. Using a well studied tritrophic food chain model in ecology as an illustrative example, we suggest two approaches to accomplish this. In the first, the overall degree of structural sensitivity in the system is treated as a function of the error terms in each function, and then we compute its gradient weighted by the local error terms. In the second, each single function in turn is allowed to vary, and we compute the degree of sensitivity when the others are kept fixed. We then take the average of this sensitivity across the space of values of the fixed functions to arrive at a form of partial degree of structural sensitivity. The partial degrees of sensitivity measure the total contribution of each function to model output uncertainty and are analogous to the total sensitivity index in the Sobol–Jansen framework^29^. In order to illustrate how the approach can be used, we propose a generic procedure of sensitivity analysis, experimentation and model refinement through which the structural sensitivity of a model can be iteratively reduced.

## Methods

### Quantifying structural sensitivity in models with uncertain component functions

In general, we consider a system of the form:

1 $$\begin{aligned} \dot{{\mathbf{x }}}={\mathbf{G}} \left( h_1\left( {\mathbf{x}} \right) ,h_2\left( {\mathbf{x}} \right) ,\ldots ,h_p\left( {\mathbf{x}} \right) , f_1\left( {\mathbf{x}} \right) ,f_2\left( {\mathbf{x}} \right) ,\ldots ,f_{m-p}\left( {\mathbf{x}} \right) \right) , \end{aligned}$$

where ${\mathbf {x}}\in {\mathbb {R}}$ is the vector of *d* state variables, $h_i, f_i:{\mathbb {R}}^{d_i}\rightarrow {\mathbb {R}}$ are the *m* different component functions describing the inflows and outflows of biomass, energy or individuals due to certain biological processes, with ${\mathbf{G}} :{\mathbb {R}}^m\rightarrow {\mathbb {R}}^d$ being a composition function describing the general topology of the system. We consider that the precise mathematical formulation of the functions $f_i$ are known (or at least postulated) with the only related uncertainty being the precise choice of their parameters. The functions $h_1,\ldots h_p$ are considered to have unspecified functional form. Instead, they are represented by bounds on their derivatives matching the qualitative properties we would expect from such a function. For example, the per-capita reproduction rate of a population is generally decreasing, at least at large population numbers, while a feeding term described by a Holling type II functional response of a predator should be increasing and decelerating. The $h_i$ may also have quantitative bounds on their values:

2 $$\begin{aligned} h_i^{\text {low}}\left( {\mathbf{x}} \right)<h_i\left( {\mathbf{x}} \right) <h_i^{\text {upp}}\left( {\mathbf{x}} \right) , \end{aligned}$$

which can be either hypothetical, or obtained from experimental fitting.

In the context of functions fitted to experimental data on biological processes, it often makes sense to consider these upper and lower bounds to be given by functions a given absolute or relative distance from a fitted function $\hat{h}_i$, which we refer to as the ‘base function’. For instance, considering a maximal absolute error of $\varepsilon _i$ gives us

3 $$\begin{aligned} h_i^{\text {low}}\left( {\mathbf{x}} \right)&:=\hat{h}_i\left( {\mathbf{x}} \right) -\varepsilon _i, \end{aligned}$$

4 $$\begin{aligned} h_i^{\text {upp}}\left( {\mathbf{x}} \right)&:=\hat{h}_i\left( {\mathbf{x}} \right) +\varepsilon _i. \end{aligned}$$

To obtain a concrete biological model from system (1), we need to specify precise equations for the uncertain functions $h_i$, as well as specifying parameters for both the $f_i$ and our specific choices of $h_i$. Without any additional information, our choice of equation to represent a given $h_i$ is essentially arbitrarily taken from the infinite-dimensional space of possible functions which satisfy (2) along with the qualitative restrictions on the derivatives. If we vary the parameters of this choice of functions, we can only cover a finite dimensional subset of this infinite dimensional space with the exact dimension of the subset determined by the number of parameters in the chosen functions. However, if we wish to fully investigate the model dynamics that can be exhibited by the system, we need to somehow consider the whole of this infinite-dimensional space. Previously the authors have developed an approach to do this with respect to the linear stability of (hyperbolic) equilibria^3,4^ by using the fact that this is determined by the eigenvalues of the Jacobian matrix of the system at the equilibrium^30^, which only depends upon the equilibrium values ${\mathbf{x}} ^*$, the values of the unknown functions at this equilibrium $h_{i}\left( {\mathbf{x}} ^{*}\right)$ and their partial derivatives $\frac{\partial h_i}{\partial x_j}\left( {\mathbf{x}} ^{*}\right)$ (along with the parameters of the fixed model functions). If we are able to adequately project the space of valid model functions into this finite-dimensional space, then we can not only check for the case that different mathematical formulations of functions can give conflicting predictions for stability, but can also quantify the likelihood of stability or otherwise by comparing the size of the volumes of the regions giving each type of behaviour.

Projecting the infinite-dimensional space of valid functions onto the finite-dimensional space of values that determine the Jacobian is the core of this approach to quantify structural sensitivity^3,4^. First, we restrict the functions $h_i$ to those with Lipschitz continuous first derivatives described by the maximal Lipschitz constants $A_i>0$. This implies that

5 $$\begin{aligned} \sum \limits _{k=1}^{n} \left|\frac{\partial ^2 h_i}{\partial x_j \partial x_k} \left( {\mathbf{x}} \right) \right|< A_i, \quad \forall j\in \left\{ 1,\ldots p \right\} . \end{aligned}$$

The question of projection can now be stated more precisely as follows: given the set of local values ${\mathbf{x}} ^*, h_i({\mathbf{x}} ^*)$, and $\frac{\partial h_i}{\partial x_j}\left( {\mathbf{x}} ^*\right)$, does at least one set of functions $h_1,\ldots ,h_p$ exist which takes these values while the $h_i$ both fit the base function $\hat{h}_i$ adequately [by satisfying (2)] and satisfy their respective qualitative restrictions (monotonicity, deceleration etc.) and maximal Lipschitz constants stated in (5).

#### ***Remark***

The introduction of maximal Lipschitz constants on the first derivatives is necessary because of the nature of the bounds (2). These bound the values of $h_i$ directly, to within a given tolerance of a fitted base function, which entails the use of a $C^0$-metric. Without further bounds on the second derivatives of the functions, the first derivatives of the functions (and consequently, the Jacobian of the system) can be perturbed to any degree through an arbitrarily small perturbation in the $C^0$-metric. This permits nonsensical outcomes, such as the appearance of an arbitrarily large number of equilibria in a slight perturbation of a system with a single equilibrium (see Section 2.5 of Kuznetsov^30^ for further explanation). For this reason, the most common types of metric used in dynamical systems are $C^1$-metrics, which measure the distance between two functions based on the sum of distance between the two functions and the distance between their first derivatives. For the current application, the use of a $C^1$ metric would force the first derivatives of functions to remain close to the first derivatives of the base function, which raises the question of how we can justify the first derivatives of the fitted base function. In the best case, data on the first derivatives of function is available, as well as data on their values, so that we can choose a base function which fits both data sets. However, the availability of such data (or data of high-enough quality that the derivatives can be reliably estimated) is extremely rare, especially in the life sciences. The introduction of Lipschitz constants on the first derivatives provides an alternative way to restrict variation of the derivatives: on the space of functions with Lipschitz continuous first derivatives of a given Lipschitz constant, the $C^0$-metric is topologically equivalent to the $C^1$ metric.

The two sets of requirements for each function yield two sets of upper and lower bounds for each $h_i$. One set is given directly by the error bounds $h_i^{\text {upp}}\left( {\mathbf{x}} \right)$ and $h_i^{\text {low}}\left( {\mathbf{x}} \right)$ specified in (2). The second set is constructed by using the bounds on the second derivatives following from the maximal Lipschitz constant of the first derivatives (5), and the qualitative restrictions on the first and second derivatives to derive tangent curves at ${\mathbf{x}} ^*, h_i({\mathbf{x}} ^*)$, $h_i^{'}\left( {\mathbf{x}} ^*\right)$: an upper tangent curve $u_i\left( {\mathbf{x}} \right)$ and a lower one $l_i\left( {\mathbf{x}} \right)$. Previously, it was shown^4^ that the following conditions are necessary and sufficient for the existence of a valid function *h* depending on a single variable *x* (here we drop the indices *i* and *j* for clarity) for a wide class of qualitative restrictions:

6 $$\begin{aligned} u (x)&> h^{\text {low}} (x) \quad \forall x \in \left[ x_{\text {min}},x_{\text {max}}\right] , \end{aligned}$$

7 $$\begin{aligned} l (x)&< h^{\text {upp}} (x) \quad \forall x \in \left[ x_{\text {min}},x_{\text {max}}\right] . \end{aligned}$$

Namely, if the set of ‘tangent’ upper and lower bounds at $\left( x^*,h\left( x^*\right) ,h^{'}\left( x^*\right) \right)$ are consistent with the quantitative bounds on the functions, then there exists a valid function within the quantitative bounds, satisfying the qualitative restrictions and passing through $\left( x^*,h\left( x^*\right) \right)$ with slope $h^{'}\left( x^*\right)$. The same logic can be applied to the more general case of scalar functions with vector inputs, $h\left( {\mathbf{x}} \right) ,\; {\mathbf{x}} \in {\mathbb {R}}^n, n>1$.

The set of values ${\mathbf{x}} ^*, h_i\left( {\mathbf{x}} ^*\right) , h'_i\left( {\mathbf{x}} ^*\right) ,\;i=1,\ldots ,p,$ which satisfy the above conditions form a closed region $V\subset {\mathbb {R}}^{n+p+np}$ which corresponds to all sets of valid functions $h_i$ (note that *V* will only be $n+p+np$-dimensional if all of the *p* unknown functions depend on all of the *n* variables of the dynamical system, and absolutely none of the values of the equilibrium or the functions at that equilibrium can be obtained from the the isocline equations of the system). By computing the eigenvalues of the Jacobian matrix determined by points in *V*, it can be subdivided into regions $V_{\text {stable}}$ and $V_{\text {unstable}}$ in which the corresponding equilibrium is stable and unstable, respectively. With the introduction of a probability density function $\rho :V\Rightarrow {\mathbb {R}}^+$, we can consider the probability of an equilibrium being stable/unstable, and that of two different choices of function yielding conflicting predictions. Since this ranges from 0 to 0.5 in the case of minimal and maximal sensitivity, respectively, we multiply it by two to get the (total) degree of structural sensitivity, given by the following definition:

#### **Definition 1**

The degree of structural sensitivity of a set of local function values $V\subset {\mathbb {R}}^{n+p+np}$, with a given probability density function $\rho :V\Rightarrow {\mathbb {R}}^+$ is given by

8 $$\begin{aligned} \Delta :=4 \cdot \int _{V_{\text {stable}}} \rho \, dV \cdot \int _{V_{\text {unstable}}} \rho \, dV = 4 \cdot \int _{V_{\text {stable}}} \rho \, dV \cdot \left( 1 - \int _{V_{\text {stable}}} \rho \, dV \right) . \end{aligned}$$

The degree of sensitivity $\Delta$ takes values between 0, when the functions either all yield a stable equilibrium or all an unstable equilibrium, and 1, when half of the functions yield a stable equilibrium and the other half an unstable equilibrium, and the model essentially gives us no information about the equilibrium’s stability. It quantifies the structural sensitivity in the system by capturing the uncertainty in the model output as a result of the uncertainty in the model functions, and can be used to determine how the structural sensitivity of a system depends on the model parameters.

The above definition allows two interpretations of uncertainty. (i) The most straightforward interpretation is that $\Delta$ is proportional to the probability that the stability of the given equilibrium will be different for two independent choices of the uncertain model functions. (ii) To interpret $\Delta$ in terms of the variance of model outputs, which is conventionally used in uncertainty and sensitivity analysis, we consider the model output *Y* to be the Bernoulli process corresponding to the stability/instability of the equilibrium for a randomly chosen set of functions. Then we have $\Delta =4\cdot {\text {Var}}(Y)$ (the scaling by 4 simply ensures that $\Delta$ ranges between 0 and 1) From (ii), it is clear that computation of the degree of structural sensitivity is a form of uncertainty analysis, in that we compute the variance of a model output (in the sense of the stability of an equilibrium) when all of the unknown functions may vary across their entire valid range.

One open question concerns which probability density function $\rho$ to consider on the space of local function values. A uniform distribution may be the most natural case to reflect the fact that we have little direct information about the distribution of points in this space. In this case, $\Delta$ will be expressed purely in terms of the relative volume of *V* for which the equilibrium is stable. Alternatively, if the error terms in the functions are assumed to be normally distributed around their respective base functions, we may construct a ‘layer cake’ approximation to the corresponding probability distribution in *V* by considering successively smaller error terms converging on the base function, and computing the corresponding regions of local function values that are valid for each. We can then assign a probability density to these values according to the size of the error term for which they still correspond to a valid function^3^.

### Two approaches to quantify the relative contribution of each function to uncertainty

In the case of a single unknown function in the model ($p=1$), the degree of structural sensitivity gives us a full analysis of the structural sensitivity in the system (possibly in combination with some parameter-based sensitivity analysis). However, in the more likely case that multiple functions are unknown ($p>1$), an important question remains: which of the unknown functions contribute the most to the degree of structural sensitivity in the system? The degree of structural sensitivity does not distinguish between the various sources of uncertainty and therefore cannot quantify the relative contributions of the unknown functions to the uncertainty in the model dynamics.

To determine the contribution of each unknown function $h_i$, one can allow the error terms $\left( \varepsilon _1,\varepsilon _2,\ldots ,\varepsilon _p\right)$ to vary with the goal of investigating how the degree of sensitivity varies with them. For the purpose of this section, let us denote the initial error terms by $\varepsilon _i^0$. We might be tempted to use the dependence on the $\varepsilon _i$ to perform global optimisation under certain constraints to find the best possible reduction of $\left( \varepsilon _1,\varepsilon _2,\ldots ,\varepsilon _p\right)$. However, one should bear in mind that this analysis would depend on the base functions $\hat{h}_i$ considered. While these functions are ideally fitted to experimental data, they are only accurate within the error terms $\varepsilon _i^0$. Excessively reducing the $\varepsilon _i$ will force all admissible functions to conform strongly in their shape to these base functions far beyond their demonstrated accuracy of fit.

The dependence of the degree of sensitivity on $\varepsilon _i$ should therefore only be evaluated *locally* by calculating the gradient $\left( \frac{\partial \Delta }{\partial \varepsilon _1},\ldots ,\frac{\partial \Delta }{\partial \varepsilon _p}\right) |_{\left( \varepsilon _1^0,\ldots \varepsilon _p^0\right) }$ giving the direction for the best local reduction of the errors. To adjust for the fact that the error terms may be of different orders of magnitude, when handling the vectors of error terms we should use the norm

9 $$\begin{aligned} \left\| \varvec{\varepsilon }\right\| = \sqrt{\sum _{i=1}^{p} \left( \frac{\varepsilon _i }{\varepsilon _i^0}\right) ^2}. \end{aligned}$$

Working in this norm, the gradient needs to be weighted by the initial error terms to provide the direction for the best local reduction of the error terms, this is described by the following structural sensitivity gradient.

#### **Definition 2**

The structural sensitivity gradient in a model with *p* unknown functions each having an error of magnitude $\varepsilon ^0_i$ is defined as

10 $$\begin{aligned} \left( -\varepsilon _1^0\cdot \frac{\partial \Delta }{\partial \varepsilon _1},\ldots ,-\varepsilon _p^0\cdot \frac{\partial \Delta }{\partial \varepsilon _p}\right) |_{\left( \varepsilon _1=\varepsilon _1^0,\ldots ,\varepsilon _p=\varepsilon _p^0\right) }, \end{aligned}$$

where $\Delta \left( \varepsilon _1,\ldots ,\varepsilon _p\right)$ is the degree of structural sensitivity of the system considered as a function of the error terms $\varepsilon _i$.

One possible problem with the structural sensitivity gradient is that the degree of structural sensitivity in the system may not be an increasing function of the magnitude of the errors. Consider the case that the exact system is structurally unstable, e.g. at a bifurcation point. Then no matter how small the error terms are, there may still be very high levels of structural sensitivity, while larger error terms may cause the level of uncertainty to decrease^5^. In this case, the structural sensitivity gradient will indicate that one or more of the functions has a negative contribution to the uncertainty of the system, and cannot be taken as a basis for sensitivity analysis.

An alternative approach to quantifying the individual impact of unknown functions which avoids this issue is the computation of partial degrees of sensitivity with respect to each $h_k$. To do this, we fix every unknown function except $h_k$, a set which we denote ${\mathbf{H}} _{\sim k}$, by fixing the $x_j^*$, $h_i\left( {\mathbf{x}} ^*\right)$, and $\frac{\partial h_i}{\partial x_j} \left( {\mathbf{x}} ^* \right)$ that are consequently determined by the isocline equations. Denoting by $V_k$ the cross-sections of *V* where only $h_k$ varies, and the cross-sections for ${\mathbf{H}} _{\sim k}$ by $V_{\sim k}$, the local partial degree of structural sensitivity can be defined as follows.

#### **Definition 3**

The local partial degree of structural sensitivity with respect to $h_k$, is the degree of structural sensitivity in the model when $h_k$ is unspecified and all other functions $h_i \in {\mathbf{H}} _{\sim k}$ are fixed:

11 $$\begin{aligned} \Delta _k({\mathbf{H}} _{\sim k}):= 4 \cdot \int _{V_{k_{\text {stable}}}} \rho _{\mathbf{H} _k\vert {\mathbf{H}} _{\sim k}} \, d{\mathbf{H}} _k \cdot \left( 1 - \int _{V_{k_{\text {stable}}}} \rho _{\mathbf{H} _k\vert {\mathbf{H}} _{\sim k}} \, d{\mathbf{H}} _k \right) , \end{aligned}$$

where $\rho _{\mathbf{H} _k\vert {\mathbf{H}} _{\sim k}}$ is the conditional probability density function on ${\mathbf{H}} _{\sim k}$:

$$\begin{aligned} \rho _{\mathbf{H} _k\vert {\mathbf{H}} _{\sim k}} = \frac{\rho }{\int _V \rho d{\mathbf{H}} _{\sim k }}, \end{aligned}$$

with $\rho$ the (joint) probability distribution over *V*.

The local partial sensitivity $\Delta _k({\mathbf{H}} _{\sim k})$ is a function of ${\mathbf{H}} _{\sim k}$ in that it depends upon the particular values at which the elements of $V_{\sim k}$ are fixed. As with the degree of structural sensitivity, it can be interpreted as either the probability that the stability of the given equilibrium will be different for two independent choices of the function $h_k$ when the $h_{\sim k}$ are fixed at the given values, or in terms of variance as $\Delta _k({\mathbf{H}} _{\sim k})=4\cdot {\text {Var}}_k(Y\vert {\mathbf{H}} _{\sim k})$ (*Y* is the Bernoulli variable for stability). If the joint probability distribution $\rho$ is uniform in *V*, then $\Delta _k({\mathbf{H}} _{\sim k})$ can be expressed purely in terms of the fraction of the volume of $V_k$ which gives a stable equilibrium:

12 $$\begin{aligned} \Delta _k({\mathbf{H}} _{\sim k}) = 4 \cdot \frac{\int _{V_{k_{\text {stable}}}} d{\mathbf{H}} _k}{\int _{V_k} d{\mathbf{H}} _k} \cdot \left( 1 - \frac{\int _{V_{k_{\text {stable}}}} d{\mathbf{H}} _k}{\int _{V_k} d{\mathbf{H}} _k} \right) . \end{aligned}$$

To obtain a global measure for the sensitivity of the model to $h_k$, we can take the average of $\Delta _k$ over $V_{\sim k}$.

#### **Definition 4**

The partial degree of structural sensitivity with respect to $h_k$ is given by

13 $$\begin{aligned} \bar{\Delta }_k := \int _{V_{\sim k}} \rho _{\mathbf{H} _{\sim k}} \cdot \Delta _k({\mathbf{H}} _{\sim k}) \, d{\mathbf{H}} _{\sim k} \end{aligned}$$

where $\rho _{\mathbf{H} _{\sim k}}$ is the marginal probability density function of ${\mathbf{H}} _{\sim k}$.

Recalling the variance-based interpretation of the degree of sensitivity, we obtain $\bar{\Delta }_k = 4\cdot E_{\sim k}({\text {Var}}_k\left( Y\vert {\mathbf{H}} _{\sim k}\right) )$. In other words, $\bar{\Delta }_k$ gives the scaled average variance when all functions except $h_k$ are fixed. We can also relate the partial degree of structural sensitivity to indices used in conventional variance-based sensitivity analysis. Dividing $\bar{\Delta }_k$ by the overall degree of structural sensitivity in the model gives us $\frac{\bar{\Delta }_k}{\Delta }=\frac{E_{\sim k}({\text {Var}}_k\left( Y\vert {\mathbf{H}} _{\sim k}\right) )}{{\text {Var}}(Y)}=S_{T_k}$, the total effect index^23^ of $h_k$ on the stability of the equilibrium. This is a measure of the total contribution of $h_k$ to the sensitivity—both alone and in conjunction with the other functions ${\mathbf{H}} _{\sim k}$. However, since the space of valid functions *V* is in general not a hypercube, the functions $h_i$ are not independent factors, and a total decomposition of variance is not possible. Indeed, even if the joint probability distribution $\rho$ is uniform, the marginal probability distribution $\rho _{\mathbf{H} _{\sim k}}$ will generally not be: instead it will equal the volume of the corresponding cross-section $V_k$ for ${\mathbf{H}} _{\sim k}$, divided by the volume of *V*. An alternative to using the partial degrees of sensitivity would be to consider the first-order sensitivity indices $S_k=\frac{{\text {Var}}_k\left( E_{\mathbf{H} _{\sim k}}\left( Y\vert h_k \right) \right) }{{\text {Var}}(Y)}$. However, these do not take into account possible joint effects of the $h_i$ on the structural sensitivity of the system, so a small $S_k$ does not indicate that $h_k$ is not a source of sensitivity, whereas $\bar{\Delta }_k=0$ means that $h_k$ does not contribute to the structural sensitivity in the system at all.

Similarly to the gradient of the total degree of sensitivity $\Delta$ as a function of the respective error tolerances, the vector $\left( -\bar{\Delta }_{h_1},\ldots , -\bar{\Delta }_{h_p}\right)$ needs to be scaled by the elements of $\varepsilon ^0$ to give us the optimal direction of decrease in $\Delta$ if the error terms $\varepsilon _i$ are subject to a proportional reduction. This is described by $\left( -\varepsilon _1^0 \bar{\Delta }_{h_1},\ldots , -\varepsilon _p^0\bar{\Delta }_{h_p}\right)$.

### Outline of an iterative framework of experiments for reducing structural sensitivity

When dealing with partially specified models, an important practical task is the reduction of the overall uncertainty in the system by decreasing the uncertainty in the system processes (i.e. the unknown model functions). Here we propose an iterative process of such a reduction based on improving our empirical knowledge of the uncertain functions $h_k$.

As a starting point, we assume that experiments have produced data on the unknown functions $h_1,\ldots ,h_p$, to which we can fit some base functions $\hat{h}_1,\ldots ,\hat{h}_p$ with initial errors $\varepsilon _1^0,\ldots ,\varepsilon _p^0$. We assume that it is possible to perform additional experiments on all uncertain processes in order to obtain more data such that the $\varepsilon _i$ can be decreased, but with the natural constraint that the total error can only be reduced by a magnitude of $0<c<1$ in each round of experiments. The main question we consider here is: by which ratio should we aim to reduce the different error terms to achieve the maximum total reduction in structural sensitivity?

**Figure 1:**
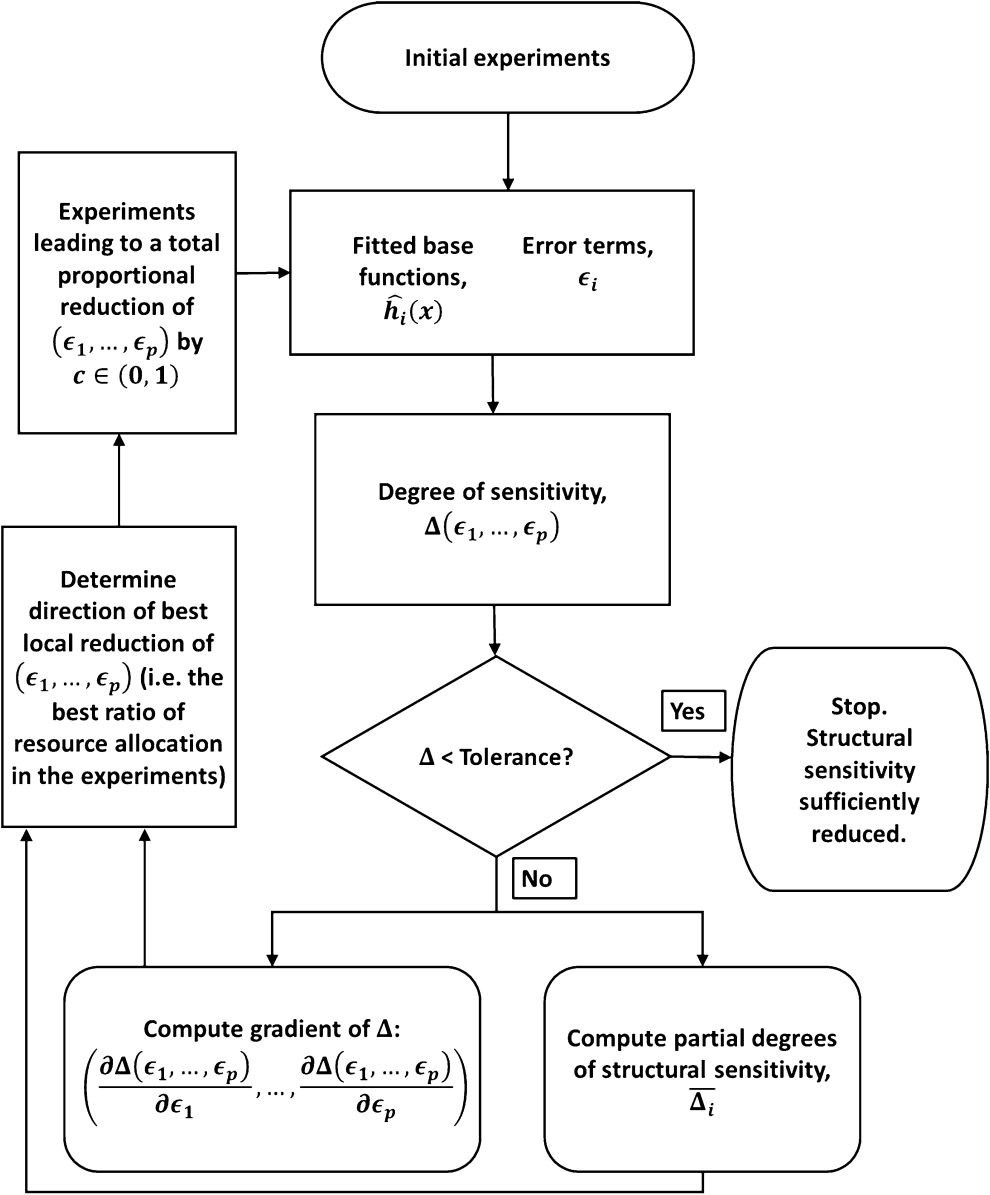
Schematic diagram of an iterative process of experiments and analysis to reduce structural sensitivity in a system with multiple uncertain functions.

The essence of the approach is as follows (an outline is shown in Fig. 1). To determine the optimal step of length *c* by which errors in the space of $(\varepsilon _1,\ldots ,\varepsilon _p)$ may be reduced, we can choose either of the approaches given in the previous section: either using the gradient of the total degree of structural sensitivity, or the ratio of the partial degrees of structural sensitivity. If the gradient is used, it can be accurately approximated using finite differences with a small step size. New experiments can then be carried out to obtain extra data to which we can fit new base functions $\hat{h}_1,\ldots ,\hat{h}_p$ with more accurate error terms $\varepsilon _1^1,\ldots ,\varepsilon _p^1$, with the aim that these new error terms should be as close as possible to those calculated to give the maximum reduction in structural sensitivity. The degree of sensitivity in the system can be computed at this stage to check that a corresponding reduction has been achieved. The process can then be repeated with the new base functions and error terms, until the structural sensitivity in the system has been reduced to an acceptable level. Note that the method resembles the gradient descent algorithm for iteratively finding local minima of a function, except with a fixed step size. In order to demonstrate the approach, in the next sections we show how structural sensitivity can be quantified and attributed to different uncertain processes in a well-known tritrophic food chain model, and apply the iterative framework to the model with a plausible artificial sequence of experiments.

## Results

### Description of a partially specified tritrophic food chain model

We consider the well-known tritrophic Rosenzweig-MacArthur food chain model introduced by Hastings and Powell^31^:

14 $$\begin{aligned} \frac{dx}{dt}&= x g\left( x\right) - \frac{a_1 x y}{b_1 + x}, \end{aligned}$$

15 $$\begin{aligned} \frac{dy}{dt}&= \frac{k_1 a_1 x y}{b_1 + x} - f\left( y\right) z - d_1 y, \end{aligned}$$

16 $$\begin{aligned} \frac{dz}{dt}&= k_2 f\left( y\right) z-d_2 z, \end{aligned}$$

where *x* is the population of a resource, *y* is an intermediate consumer and *z* is a top predator. We consider two unspecified functions: the per-capita growth rate of the resource, $g\left( x\right)$, and the functional response of the top predator, $f\left( y\right)$, the per-capita feeding rate as a function of prey density. Consumption of *x* by *y* is described by a functional response given by the Holling disc equation^7^, with maximum attack rate $a_1>0$ and half saturation constant $b_1>0$. $k_1>0$ and $k_2>0$ are trophic conversion factors and $d_1>0$ and $d_2>0$ are linear mortality rates of each predator. In the notation of the Methods section, the number of unspecified functions $p=2$, with $h_1\left( x,y,z\right) \equiv g\left( x\right)$ and $h_2\left( x,y,z\right) \equiv f\left( y\right)$. The remaining, specified model functions are the consumer functional response $f_1\left( x,y,z\right) = \frac{a_1xy}{b_1+x}$, the consumer mortality $f_2\left( x,y,z\right) = d_1 y$, and the top predator mortality $f_3\left( x,y,z\right) = d_2 z$.

**Figure 2:**
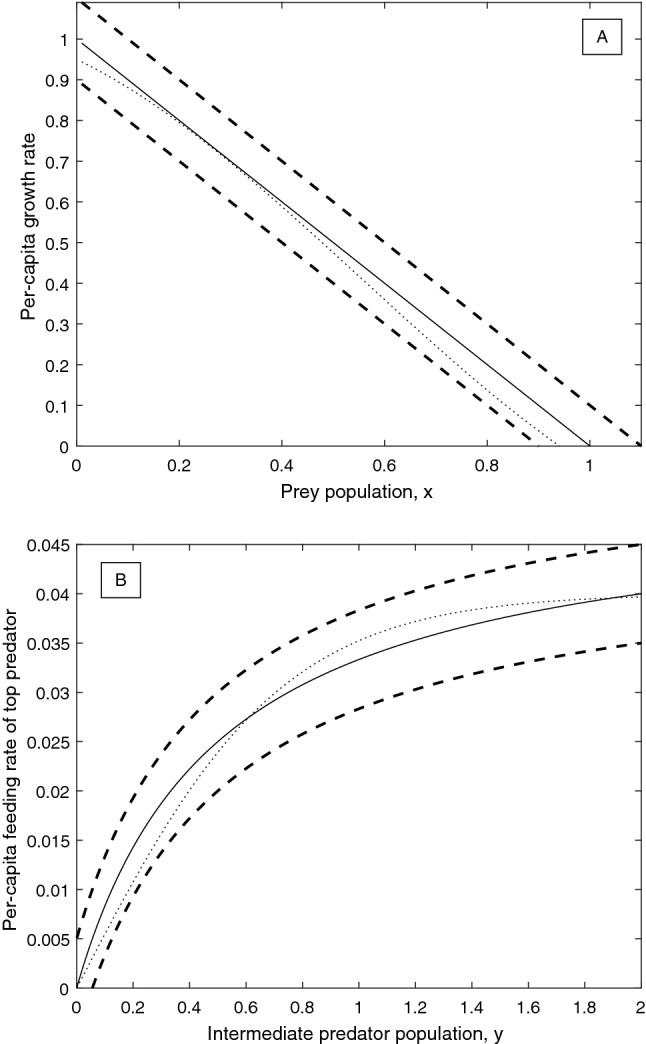
Sample base functions (solid lines) and maximal error bounds (dashed lines) for system (14)–(16). The dotted lines correspond to the correct model functions as defined in Eqs. (19) and (20) (**A**) Logistic per-capita growth rate base function (21), together with maximal error bounds for $\varepsilon _g=0.1$. (**B**) Holling type II predation base function (22), together with maximal error bounds for $\varepsilon _f=0.005$.

We fix the parameters of all specified model components at baseline values of $a_1=5$, $b_1=2$, $k_1=1$, $d_1=0.4$, $k_2=0.9$ and $d_2=0.01$. We assume that experimental data is available on the unspecified functions *g* and *f*, to which base functions of the form $\hat{g}\left( x\right) =r\left( 1-\frac{x}{K}\right)$ and $\hat{f}\left( x\right) =\frac{a_2 x}{1+b_2 x}$ can be fitted, respectively, with $r=1$, $K=1$, $a_2=0.1$, $b_2=2$. However, these fits are not perfect: the growth rate may have a maximum absolute error of $\varepsilon _g = 0.1$, and the functional response may have a maximum error of $\varepsilon _f = 0.005$. The fitted base functions are shown together with their error bounds in Fig. 2A and B. In addition to fitting available data, we also consider qualitative restrictions on the derivatives of *g* and *f*. For the functional response *f*, we require that it is of Holling type II, i.e. both increasing and decelerating, so that $f'\left( y\right) >0, f''\left( y\right) <0 \;\; \forall y \in \left[ 0,y_{ \text {max}}\right]$. Furthermore, in the absence of prey no predation is possible, so that $f\left( 0\right) =0$ must hold. Finally, as in (5), we consider that $f'$ is a Lipschitz continuous function with Lipschitz constant no greater than $B>0$, that is $|f^{''}(y)|<B \;\; \forall y \in \left[ 0,y_{ \text {max}}\right] .$ For the per-capita growth rate of the resource, we make no further qualitative restrictions, except for the necessary requirement that $g'$ is Lipschitz continuous with Lipschitz constant no greater than $A>0$ so that $|g^{''}(x)|<A \;\; \forall x \in \left[ 0,x_{ \text {max}}\right] .$

Based on the above assumptions, the resource growth rate must be a function $g\left( x\right)$ satisfying the following conditions:

(i) $\hat{g}\left( x\right) -\varepsilon _g<g\left( x\right) < \hat{g}\left( x\right) +\varepsilon _g$ : The function must lie within the error bounds (see Fig. 2A).
(ii) $|g^{''}(x)|< A \quad \forall x \in \left[ 0,x_{\text {max}}\right] , A>0$ : The growth rate function must have a Lipschitz-continuous first derivative with Lipschitz constant at most *A*. In this paper, we take $A=10$.

The functional response of the top predator must be a function $f\left( y\right)$ satisfying the following conditions:

(i) $\hat{f}\left( y\right) -\varepsilon _f<f\left( y\right) < \hat{f}\left( y\right) +\varepsilon _f$ : The function must lie within the error bounds (see Fig. 2B).
(ii) $f\left( 0\right) =0$ : The absence of prey implies the absence of predation.
(iii) $f'\left( y\right) >0 \quad \forall y \in \left[ 0,y_{ \text {max}}\right]$ : Per-capita predation rate is an increasing function of prey density.
(iv) $-B<f''\left( y\right) <0 \quad \forall y \in \left[ 0,y_{ \text {max}}\right]$ : Per-capita predation rate is a decelerating function of prey density, and the functional response must have a Lipschitz-continuous first derivative with Lipschitz constant at most *B*. In this paper, we take $B=10$.

Any coexistence equilibria of the system must satisfy the isocline equations (Supplementary Material Appendix A). For any strictly increasing function *f*, a single equilibrium value $y^*$ for the intermediate predator is possible. Even if *g* is considered to be strictly increasing, multiple equilibrium values for the lowest trophic species $x^*$, and consequently multiple $z^*$ values, are possible, but not for the parameters considered here.

### Quantifying the structural sensitivity of the model

Without specifying the functions *f* or *g*, we cannot determine the equilibria of the system or their stability, but a full description of the functions *f* and *g* is not necessary for this: only four values are. To fully determine the equilibrium values in the system, it is enough for us to know the local values $y^*$ and $g\left( x^*\right)$. The linear stability of a given equilibrium is determined by the linearisation of the system at this equilibrium, represented by the Jacobian matrix (Supplementary Material Appendix A)), for which we also need to know the quantities $g'\left( x^*\right)$ and $f'\left( y^*\right)$. All other necessary values are fixed by the isocline equations. The question of whether or not the stability of the equilibrium is sensitive to the precise equations for *f* and *g* is therefore equivalent to the question of whether or not the set *V* of values $\left( y^*, g\left( x^*\right) , g'\left( x^*\right) , f'\left( y^*\right) \right) \in {\mathbb {R}} ^4$ which can be taken by valid functions *g* and *f* (i.e. satisfying properties (i)–(ii) and (iii)–(iv), respectively) contains subsets of positive measure which yield Jacobians with different stability properties. Essentially the problem reduces to finding a projection $\phi$ between the space of valid functions *f*, *g*, and the space of local values $\left( g\left( x^*\right) , g'\left( x^*\right) , y^*, f'\left( y^*\right) \right) \in {\mathbb {R}} ^4$ taken by such functions. The question of projection can be stated as follows: given a set of values $y^*, f(y^*), f'\left( y^*\right)$, $x^*, g\left( x^*\right) , g'\left( x^*\right)$, does there exist at least one pair of functions *f* and *g* satisfying restrictions (i)–(iv) taking these values? The conditions for the existence of such functions are found in full in^4^. Aside from some small additions concerning the other qualitative restrictions, the conditions for the existence of a valid function *g* are

17 $$\begin{aligned} u_g(x)&> g^{\text {low}}(x) := \hat{g} \left( x\right) -\varepsilon _g \quad \forall x \in \left[ 0,x_{\text {max}}\right] , \end{aligned}$$

18 $$\begin{aligned} l_g(x)&< g^{\text {upp}}(x) := \hat{g} \left( x\right) +\varepsilon _g \quad \forall x \in \left[ 0,x_{\text {max}}\right] . \end{aligned}$$

The conditions for the existence of a valid function *f* are similar. One can also use a computational method based on optimal control theory^32^ to approximately check for the existence of valid functions *f* and *g*.

**Figure 3:**
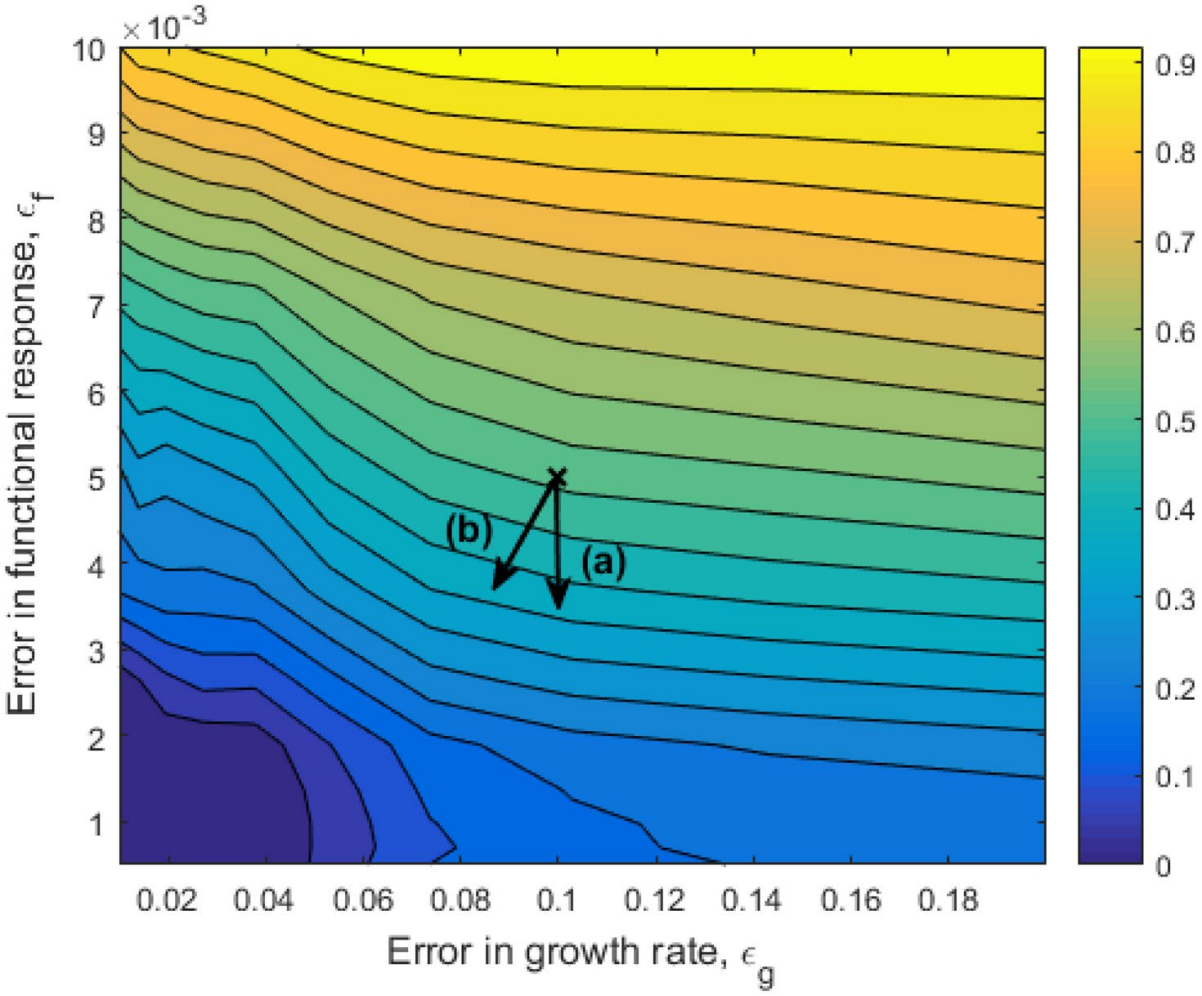
The dependence of the degree of sensitivity in system (14)–(16) on the error terms in the per-capita growth rate of the species on the lowest trophic level, $\varepsilon _g$ and the functional response of the top predator, $\varepsilon _f$. The cross marks the initial error as shown in Fig. 2A and B. (**a**) is the vector of magnitude 0.3 in the direction opposite to the gradient in the weighted norm: $\left( - \varepsilon _g^0 \cdot \frac{\partial \Delta }{\partial \varepsilon _g} , - \varepsilon _f^0 \cdot \frac{\partial \Delta }{\partial \varepsilon _f} \right)$. (b) is the vector of the same magnitude in the direction $\left( -10,-1\right)$. The standard Euclidean norm of vector (**b**) is almost 8 times that of (**a**).

The dependence of the degree of structural sensitivity of the system on the error terms $\varepsilon _g$ and $\varepsilon _f$ is shown in Fig. 3. Here most of the structural sensitivity in the system seems to be caused by uncertainty in the functional response term: reducing the error in the growth term does not reduce the uncertainty in the system without a simultaneous reduction of the error in the functional response. The weighted gradient of the degree of sensitivity, giving the direction for the best local reduction of the error terms is $\left( - \varepsilon _g^0 \cdot \frac{\partial \Delta }{\partial \varepsilon _g} , - \varepsilon _f^0 \cdot \frac{\partial \Delta }{\partial \varepsilon _f} \right) =(0.05960, 0.3319)$ (see vector (a) in Fig 3). The importance of using the weighted norm is emphasized by considering as an example the vector of the same magnitude in the weighted norm, 0.3, but in the direction $(-10,-1)$ (see vector (b) in Fig 3). In the Euclidean norm, (b) is almost 8 times as large as (a).

**Figure 4:**
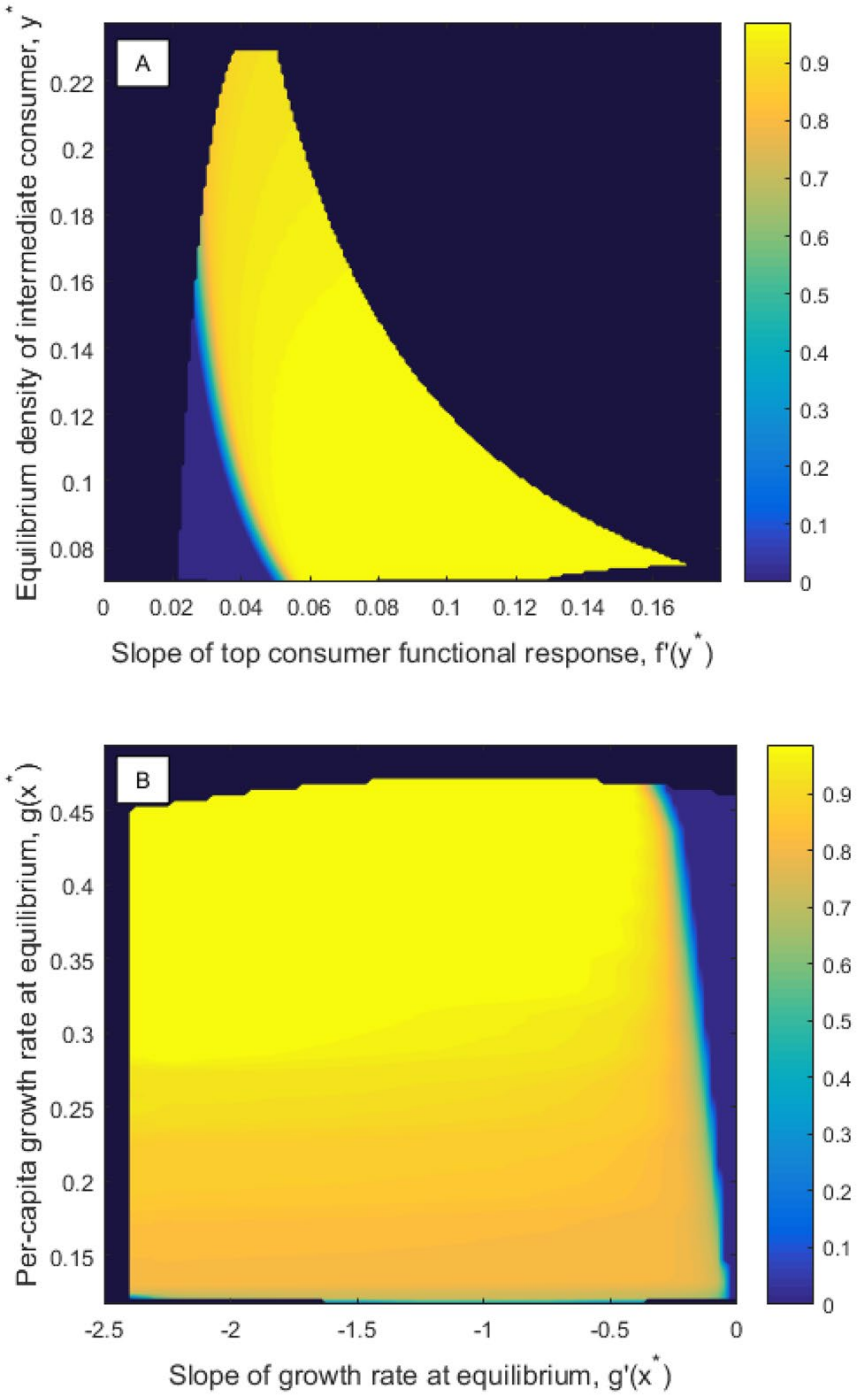
The probability of a stable nontrivial equilibrium in model (14)–(16) conditional on each unspecified function for maximal errors $\varepsilon _g=0.1$, $\varepsilon _f=0.005$, as depicted in Fig. 2. Dark blue regions lie outside of the set of valid functions, *V*. (**A**) $P\left( {\text {Stable}}\vert f \right)$ as a function of the local values of the fixed functional response *f*. Only the growth rate *g* can vary. (**B**) $P\left( {\text {Stable}}\vert g \right)$ as a function of the local values of the fixed growth rate *g*. Only the functional response *f* can vary.

In "Two approaches to quantify the relative contribution of each function to uncertainty" section, computation of partial degrees of structural sensitivity was suggested as an alternative to the gradient of the degree of structural sensitivity. To compute the partial degree of sensitivity with respect to *g*, for example, we assume the functional response *f* is fixed then compute the set of $g(x^*)$ and $g'(x^*)$ values which correspond to valid functions *g* given these fixed *f* values, a 2D cross-section $V_f$ of the 4D set *V*. Based on this cross-section, we can compute the probability that the equilibrium is stable conditional on a given *f*, and the local partial degree of structural sensitivity with respect to *g*, $\Delta _g\left( y^*, f'\left( y^*\right) \right)$. The partial sensitivity with respect to *f*, $\Delta _f$, is computed in the same way, except that in this case we fix *g*. Figure 4 shows how the probability of a stable nontrivial equilibrium in system (14)–(16) varies when each functions are fixed, with error terms $\varepsilon _g=0.1$, $\varepsilon _f=0.005$, as in Fig. 2. Corresponding plots of the local partial degree of structural sensitivity are given in Supplementary Material B: $\Delta _i=0$ when the probability of a stable equilibrium is 0 or 1, and $\Delta _i=1$ when the probability of a stable equilibrium is $\frac{1}{2}$. To obtain the partial degrees of structural sensitivity $\bar{\Delta }_g$, $\bar{\Delta }_f$, we need to take the expectation of $\Delta _g$ and $\Delta _f$ over all valid $y^*, f'\left( y^*\right)$ and $g(x^*), g'(x^*)$ values with the marginal probability distributions $\rho _g$ and $\rho _f$ (see Supplementary Material C). We find $\bar{\Delta }_g=0.2148$ and $\bar{\Delta }_f=0.3260$, indicating that the system is more sensitive overall to the functional response.

### Reducing the model structural sensitivity

To demonstrate the iterative framework for reducing model uncertainty outlined in Fig. 1, we assume that the experimental data is generated from (14)–(16) with a cubic per-capita growth rate of prey and a hyperbolic tangent functional response of the top predator:

19 $$\begin{aligned} g_{\text {True}} \left( x \right)&=\alpha _3 x^3+\alpha _2 x^2+\alpha _1 x+ \alpha _0, \end{aligned}$$

20 $$\begin{aligned} f_{\text {True}} \left( y \right)&=a_2^{\text {Trig}} \tanh {\left( b_2^ {\text {Trig}}y\right) } , \end{aligned}$$

where $\alpha _3=0.6$, $\alpha _2=-1$, $\alpha _1=-0.6$, $\alpha _0=0.95$, $a_2^{\text {Trig}}=0.04$, and $b_2^{\text {Trig}}=1.38$. In place of empirical data, we will directly consider plausible fitted base functions and error terms (chosen such that the true functions are within the given error bounds) to represent the results of experiments or measurements. For the initial data available, we assume that the fitted base functions and the error bounds match the scenario presented in the Methods section. That is, the functional response and the per-capita growth rate are given by a Holling type II function and logistic growth, respectively:

21 $$\begin{aligned} \hat{g}_0(x)&:=r\left( 1-\frac{x}{K}\right) , \end{aligned}$$

22 $$\begin{aligned} \hat{f}_0(y)&:=\frac{a^{\text {Holling}}_2 y}{1+b^{\text {Holling}}_2 y}, \end{aligned}$$

where $r=1$, $K=1$, $a^{\text {Holling}}_2=0.1$ and $b^{\text {Holling}}_2=2$. The two initial error terms are given by $\varepsilon _g^0=0.1$ and $\varepsilon _f^0=0.005$. The true functions, along with the initial base functions and error bounds are shown in Fig. 2A and B, and as was already shown, the degree of structural sensitivity in the system with these base functions and errors is $\Delta _0 \approx 0.5123$.

In order to find the best way to reduce the degree of structural sensitivity, we consider a total error reduction in each round of experiments of magnitude $c=0.3$. To determine the best direction to make this error reduction we will use the partial degrees of sensitivity. As previously shown, these are given by $\bar{\Delta }_g=0.2148$ and $\bar{\Delta }_f=0.3260$, giving us an approximate direction of optimal error decrease of $\left( -\varepsilon _g^0 \cdot \bar{\Delta }_g , -\varepsilon _f^0 \cdot \bar{\Delta }_f \right) |_{\left( \varepsilon _g=\varepsilon _g^0,\varepsilon _f=\varepsilon _f^0\right) }=\left( -0.0181,-0.00132 \right)$. A reduction in the total error of magnitude $c=0.3$ in this direction results in errors of $\varepsilon _g^1\approx 0.0835$ and $\varepsilon _f^1\approx 0.00375$.

**Figure 5:**
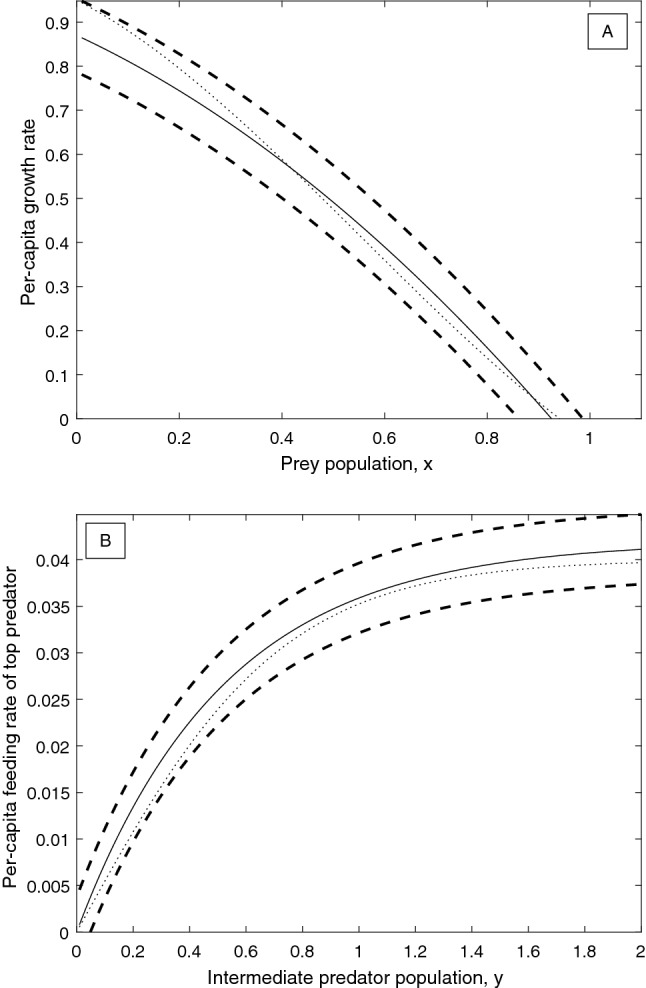
Updated base functions (solid lines) and maximal error bounds (dashed lines) after one iteration of the procedure for reducing structural sensitivity detailed in Fig. 1. (**A**) Quadratic per-capita growth rate base function (23), with maximal error $\varepsilon _g^1\approx 0.0835$. The true per-capita growth rate is given by the dotted line, for comparison. (**B**) Ivlev predation base function (24), with maximal error $\varepsilon _f^1\approx 0.00375$. The true functional response is given by the dotted line, for comparison.

**Figure 6:**
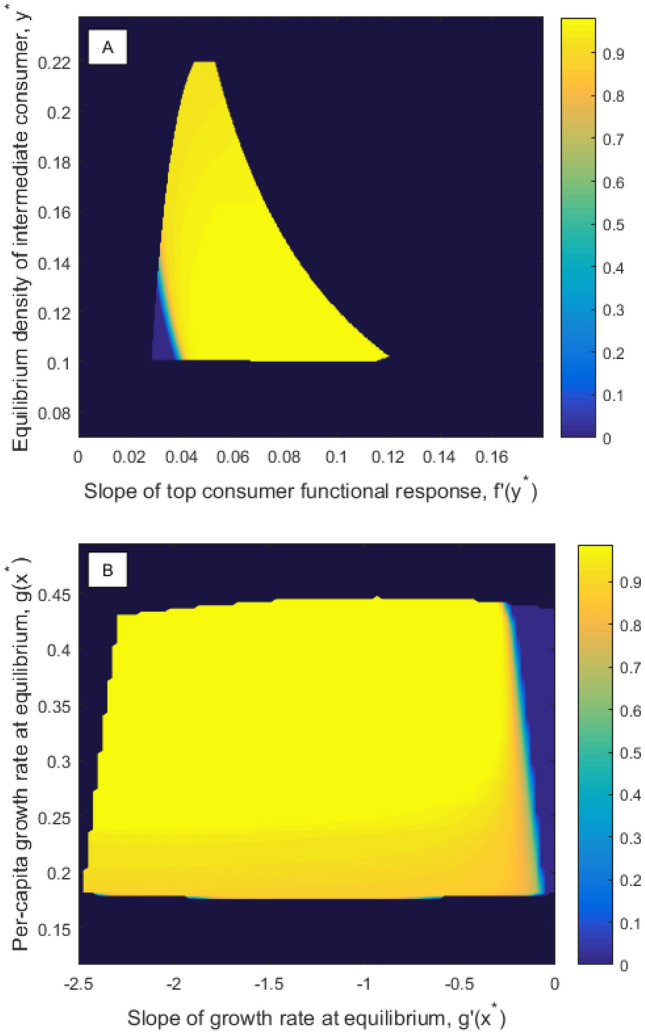
The probability of a stable nontrivial equilibrium in the model (14)–(16) conditional on each unspecified function after one iteration of the procedure for reducing structural sensitivity detailed in Fig. 1. The updated error terms are $\varepsilon _g^1\approx 0.0835$ and $\varepsilon _f^1\approx 0.00375$, as depicted in Fig. 5. Axis have the same range as in Fig. 4. Dark blue regions lie outside of the set of valid functions, *V*. (**A**) $P\left( {\text {Stable}}\vert f \right)$ as a function of the local values of the fixed functional response *f*. Only the growth rate *g* can vary. (**B**) $P\left( {\text {Stable}}\vert g \right)$ as a function of the local values of the fixed growth rate *g*. Only the functional response *f* can vary.

Consider now that a second round of experiments is conducted which yields new data on the functions such that the reduction of the errors to $\varepsilon _g^1\approx 0.0835$ and $\varepsilon _f^1\approx 0.00375$ follows. As an example, consider an Ivlev functional response and a quadratic per-capita growth function:

23 $$\begin{aligned} \hat{g}_1(x)&:=\beta _2 x^2 + \beta _1 x + \beta _0, \end{aligned}$$

24 $$\begin{aligned} \hat{f}_1(y)&:=a^{\text {Ivlev}}_2 \left( 1-\exp {b^{\text {Ivlev}}_2 y}\right) , \end{aligned}$$

where $\beta _2=-0.4303$, $\beta _1=-0.5425$, $\beta _0=0.87$ $a^{\text {Ivlev}}_2=0.042$ and $b^{\text {Ivlev}}_2=1.9269$. These updated base functions are plotted together with the corresponding error bounds and the correct functions in Fig. 5A and B. The corresponding degree of structural sensitivity in this system is found to be $\Delta _1=0.2397$, a reduction of more than 50%, substantially more than the total weighted reduction in $\varepsilon _g$ and $\varepsilon _f$ of $\approx 21\%$. In order to determine the best way to reduce the sensitivity in the system in the next round of experiments, we again compute the partial degrees of sensitivity of the system with respect to the two functions. The probability of a stable equilibrium in the system conditional on *f*, $P\left( {\text {Stable}}\vert f\right)$, is shown in Fig. 6A, and $P\left( {\text {Stable}}\vert g\right)$ is plotted in Fig. 6B. The partial degrees of sensitivity are calculated to be $\bar{\Delta }_g=0.1472$ and $\bar{\Delta }_f=0.1203$, which gives an optimal direction for error decrease of $\left( -\varepsilon _g^1 \cdot \bar{\Delta }_g , -\varepsilon _f^1 \cdot \bar{\Delta }_f \right) |_{\left( \varepsilon _g=\varepsilon _g^1,\varepsilon _f=\varepsilon _f^1\right) }=\left( -0.0123,-0.000451 \right)$, so that the optimal step of length 0.3 to reduce the error terms yields $\varepsilon _g^2\approx 0.0638$ and $\varepsilon _f^2\approx 0.00305$.

**Figure 7:**
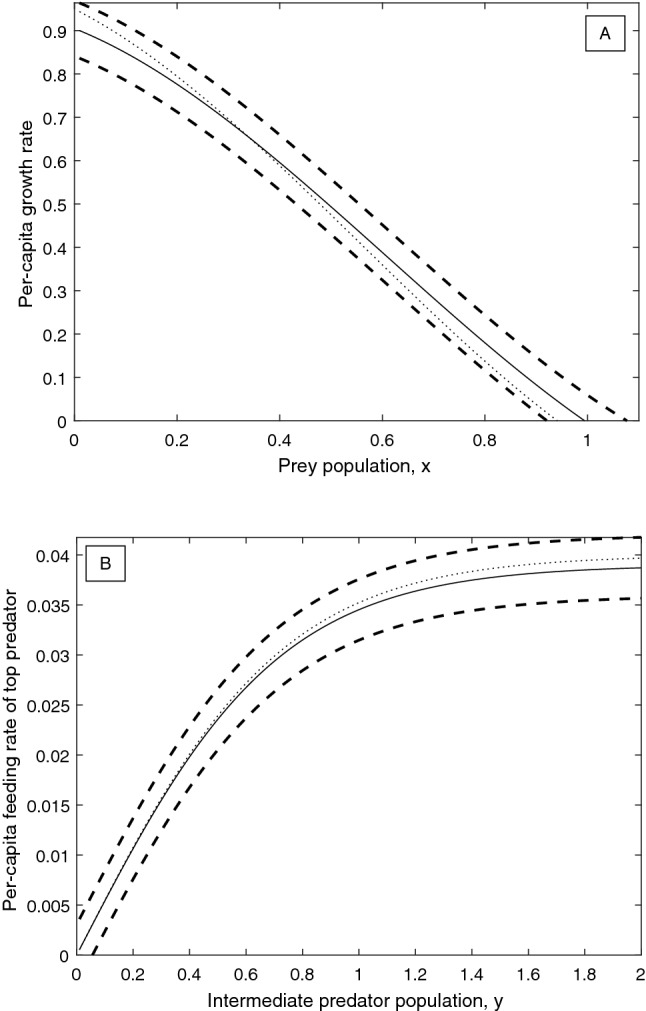
Updated base functions (solid lines) and maximal error bounds (dashed lines) after two iterations of the procedure for reducing structural sensitivity detailed in Fig. 1. (**A**) Cubic per-capita growth rate base function (25), with maximal error $\varepsilon _g^2\approx 0.0638$. The true per-capita growth rate is given by the dotted line, for comparison. (**B**) Hyperbolic tangent predation base function (26), with maximal error $\varepsilon _f^2\approx 0.00305$. The true functional response is given by the dotted line, for comparison.

Consider that a final round of experiments is conducted which yields improved data on the functional response and per-capita growth rate such that the functions can be estimated with these improved errors $\varepsilon _g^2\approx 0.0638$ and $\varepsilon _f^2\approx 0.00305$. For example, we may assume that the correct functional forms are finally fitted, but with inaccurate parameters:

25 $$\begin{aligned} \hat{g}_2(x)&=\alpha _3 x^3+\alpha _2 x^2+\alpha _1 x+\alpha _0, \end{aligned}$$

26 $$\begin{aligned} \hat{f}_2(y)&=a_2^{\text {Trig}} \tanh {\left( b_2^{\text {Trig}}y\right) }, \end{aligned}$$

where $\alpha _3=0.53$, $\alpha _2=-0.97$, $\alpha _1=-0.47$, $\alpha _0=0.905$, $a_2^{\text {Trig}}=0.039$, and $b_2^{\text {Trig}}=1.4$. These final base functions are plotted together with the new error bounds and the corresponding true functions in Fig. 7A and B. The corresponding degree of structural sensitivity in the system is found to be $\Delta _2=0.2094$. Over the course of the two iterations, the degree of structural sensitivity in the system has been reduced from $\Delta _0=0.5123$ by approximately 60%, by reducing the error terms from $\varepsilon _g^0=0.1$, $\varepsilon _f^0=0.005$, to $\varepsilon _g^2\approx 0.0638$ and $\varepsilon _f^2\approx 0.00305$, a total reduction of 38%. One may perform further iterations to continue the reduction of structural sensitivity, eventually approaching $\Delta =0$ provided the system is structurally stable; however, in practice it may be difficult to achieve an error of less than 2.5–5% in a typical biological experiment.

## Discussion

In conclusion, in this paper we address the question of sensitivity analysis of a model with respect to uncertainty in its component functions, rather than just its parameters, and we outline such an approach to sensitivity analysis where the model output we’re interested in is the stability or otherwise of an equilibrium. The consideration of uncertain functions to represent biological processes in mathematical models is necessary since the use of particular equations to represent these processes is far more restrictive than is often supposed. Models can be sensitive to changes in their constituent functions while remaining robust to variation of the parameters that specify these functions, a form of sensitivity known as structural sensitivity^1,2,13^. The structural sensitivity paradigm poses both challenges and opportunities. Parameter-based sensitivity analysis can miss much of the possible uncertainty in a model because only a severely restricted subset of the space of possible model functions is considered, and this necessitates the development of new approaches going beyond the use of precise functions. But these new approaches may also open up new prospects for identifying influential factors in biological systems and those of other disciplines^3,6,19^. We have introduced two measures to quantify the contribution of the impact of different sources of uncertainty to the model predictions for the stability of a given equilibrium state. We show how either measure can be used to inform an efficient approach to reduce the structural sensitivity in the model by iteratively obtaining targeted empirical data.

Our approach can be considered as an extension of variance-based sensitivity analysis (SA)^23^ whereby the uncertainty in the model output is partitioned among the various input parameters. The first measure we propose considers the degree of structural sensitivity in the system to depend on the error terms in each constituent model function, $\Delta \left( \varepsilon _1,\ldots ,\varepsilon _p\right)$. By varying these error terms one can approximate the gradient of the degree of structural sensitivity with respect to each of them, and the respective magnitudes of the components represent the rate of increase/decrease in model uncertainty that would result from small changes in the accuracy of the measurement of each function. Approaches to SA can be classified as either local or global. Local SA approaches revolve around computing or estimating the partial derivatives of the relevant model output with respect to each input parameter by varying one factor at a time^26^, while global SA approaches consider the effects of different inputs on the variance of the model output with sampling over the whole region of parameter values^29,33^. The gradient of sensitivity can be understood as a local SA of a global uncertainty analysis (measured by the degree of structural sensitivity) with respect to the ranges of its inputs. Note that a global SA with respect to the errors $\varepsilon _i$, or any other global exploration of this space, isn’t valid: the error bounds are obtained as confidence intervals centered around fitted base functions. Significantly smaller error bounds restrict the analysis to the particular functional forms of these base functions, which are not justified.

The second measure we introduce is based on a global SA where the input parameters are the values of the unknown functions that determine the equilibrium’s stability. These may vary over a bounded region which corresponds to model functions satisfying the necessary qualitative and quantitative constraints. We can compute the degree of sensitivity with respect to a target function when all other functions are fixed, then average this over the other functions to obtain the partial degree of sensitivity with respect to the target function, a measure of the overall sensitivity of the model to this function, including through interactions with the uncertainty in the other functions. In terms of variance-based SA, the partial degrees of sensitivity are equivalent to the total effect of each function on the stability of the equilibrium. One advantage of using the partial degrees of sensitivity over the gradient of the degree of sensitivity is that the variables determined by the general functions are treated the same as the model parameters of any fixed functions, so that sensitivity analysis with respect to the unspecified functions can be straightforwardly extended to include a simultaneous global SA with respect to the parameters of the specified functions.

Since global SA with a large number of inputs requires the evaluation of high-dimensional integrals, and the estimation of variances and expectation needs to be made based on the potentially costly computation of model outputs, techniques such as Latin Hypercube sampling^34,35^ or the use of Sobol’ sequences^29,36^ are often used to more efficiently sample the multidimensional input space in a more representative way than can be obtained by brute-force Monte Carlo approaches. In the present paper, no model evaluation is required, since only the eigenvalues of the Jacobian matrix are tested, reducing the computational demand. When the number of uncertain functions that need to be investigated are large, pseudorandom sampling strategies are likely to be beneficial. The issue is complicated, however, by the fact that the set of local function values corresponding to valid functions may have a complex geometry, with dependence among the different unspecified functions^37^, while most sampling techniques are designed for independent inputs in a hypercube.

Implementation of either measure introduced here quantifies the contribution of each unspecified function to the total structural sensitivity. This allows us to reduce uncertainty in the model outputs by suggesting an optimal ratio in which to reduce the error terms in the unspecified functions, and thus decide which experiments should best refine the model (the sketch of the procedure is shown in Fig. 1). In the tri-trophic ecosystem model considered here as an example, the structural sensitivity was reduced by around 60% from $\Delta _0=0.5123$ to $\Delta _2=0.2094$ through a reduction in the errors $\varepsilon _f$ and $\varepsilon _g$ in the unknown functions of around 40%. We argue that when designing a sequence of experiments to reduce model sensitivity, an iterative sequence of experiments should be more efficient than a single but more extensive experiment when aiming to obtain the best accuracy for unknown functions. This is because the partial sensitivities are local with respect to the error terms, and the optimal ratio in which to decrease the error terms may change as these errors are reduced and more accurate fits are obtained. However, in practice single extensive experiments may be more economical than sequences of less-extensive ones and it may only be feasible to investigate a single uncertain process at a time. In this case, the partial degrees of structural sensitivity can still be used to single out functions for further investigation. The ultimate convergence of the method is a complicated matter (if the underlying model system is structurally unstable it will be impossible), but is a moot point in practical terms, since we are rarely able to reduce error terms below a modest level due to experimental noise.

In this paper, it has been assumed that model functions are obtained directly from measurements before being introduced into the model. However, in many cases only time series data for the whole system is available, so all parts of the model needed to be fitted simultaneously by comparing model and time series outputs through methods such as maximum likelihood estimation^38,39^. With such approaches, it is possible to produce estimates of the confidence intervals of the parameters, but not of the function values themselves. To do this, advanced techniques for nonparametric regression^24,25^ may be used. Another approach is to make an initial choice of functions and determine the possible range of their parameters, then to find the maximum and minimum values for each function over this range. Except in simple cases, it will generally not be possible to maximise a given function across its entire domain simultaneously, because of non-monotonic dependence on certain parameters, or because parameters have combined effects on the likelihood. In this case, we need to decide how to specify the error bounds, whether e.g. to maximise or minimise the average of the function over its domain, to maximise each parameter simultaneously or maximise the function values piecewise, each of which has its disadvantages. Once error bounds are obtained for the function values, however, the approach outlined here can be applied as usual.

There remain several open questions with respect to the proposed structural sensitivity analysis framework. Firstly, there are situations where the method used here to compute the space of valid function values *V* is not applicable. We have projected the set of valid functions into the space of local values using arguments based on the compatibility of two sets of upper and lower bounds on functions over a 1D domain. However, we may also wish to consider functions on higher dimensional domains: for example, in model (14)–(16) we treat the functional response of a predator as a function of prey density, but it may also be dependent on predator density as with a ratio-dependent functional response. In the framework presented here, we also assume that all functions vary independently within their given range, which makes sense for independent experiments on each function, but if the functions are all estimated from the same data, modification of the projection used would be preferable. Finally, the analysis carried out here has been largely qualitative in nature, concerning the stability of an equilibrium. In general, however, other important quantitative model outputs such as non-equilibrium attractors or transient dynamics can also be sensitive to variations in the model function. All of these issues require extensions of the method, with consideration of quantitative outputs being particularly challenging since it potentially necessitates consideration of the entire range of function values.

## Supplementary information

Supplementary Information.
