# Supplementary material for: Identifying the sources of structural sensitivity in partially specified biological models
Source: Sci Rep. 2020 Oct 9;10:16926. doi: 10.1038/s41598-020-73710-z (PMC7547730; doi:10.1038/s41598-020-73710-z)
Supplement: Supplementary file 1 — Supplementary Information. [file 41598_2020_73710_MOESM1_ESM.pdf]

Supplementary Material - Identifying structural sensitivity in partially specified biological models

Matthew Adamson[1,*,+] **and Andrew Morozov**[2,3,+]

[1]University of Osnabrück, School of Mathematics, Computer Science, Osnabrück, 49076, Germany
[2]University of Leicester, Mathematics, Leicester, LE1 7RH, United Kingdom
[3]Institute of Ecology and Evolution, Russian Academy of Sciences, 33 Leninskii pr., Moscow 119071, Russia
[*]madamson@uni-osnabrueck.de
[+]these authors contributed equally to this work

A Isocline equations for the tritrophic food chain model

We consider the tritrophic Rosenzweig-MacArthur food chain model first introduced by Hastings and Powell[1]:

$$\frac{dx}{dt} = xg(x) - \frac{a_1xy}{b_1+x}, \tag{1}$$

$$\frac{dy}{dt} = \frac{k_1a_1xy}{b_1+x} - f(y)z - d_1y, \tag{2}$$

$$\frac{dz}{dt} = k_2f(y)z - d_2z,, \tag{3}$$

A summary of the parameters of all specified model components and their baseline values given by Table 1. A detailed explanation of the parameter meanings, the functions g and f and the constraints they must satisfy is provided in section 3.1 of the main text.

Table 1. Parameter meanings and baseline values

Parameter	Baseline value
Maximum attack rate of intermediate predator, a_1	5
Half-saturation constant of intermediate predator, b_1	2
Trophic conversion factor of intermediate predator, k_1	1
Mortality rate of intermediate predator, d_1	0.4
Trophic conversion factor of top predator, k_2	0.9
Mortality rate of top predator, d_2	0.01

Any coexistence equilibria in the system must satisfy the isocline equations:

$$g(x^*) = \frac{a_1y^*}{b_1+x^*},$$

$$f(y^*)z^* = \frac{k_1a_1x^*y^*}{b_1+x^*} - d_1y^*,$$

$$k_2f(y^*) = d_2,$$

which yields the following equations:

$$f(y^*) = \frac{d_2}{k_2}, \tag{4}$$

$$x^* = \frac{a_1y^* - b_1g(x^*)}{g(x^*)}, \tag{5}$$

$$z^* = \frac{k_2}{d_2}\left(k_1a_1x^*g(x^*) - d_1y^*\right). \tag{6}$$

For any strictly increasing function f, a single equilibrium value for the intermediate predator y^* is possible. Even if g is considered to be strictly increasing, multiple equilibrium values for the lowest trophic species x^* are possible, and consequently multiple z^* values. However, for the parameters considered here this is not possible.

The linear stability of a given equilibrium is determined by the linearisation of the system about this equilibrium, represented by the Jacobian matrix

$$J_{(x^*,y^*,z^*)} = \begin{pmatrix} g(x^*) + x^* g'(x^*) - \frac{b_1 g^2(x^*)}{a_1 y^*} & -\frac{x^* g'(x^*)}{y} & 0 \\ \frac{k_1 b_1}{a_1} g^2(x^*) & \frac{k_1 g(x^*) x^*}{y^*} - f'(y^*) z^* - d_1 & -\frac{d_2}{k_2} \\ 0 & k_2 f'(y^*) z^* & 0 \end{pmatrix}. \tag{7}$$

B Computation of the partial degrees of sensitivity

In this section we provide additional figures to show how the partial degrees of sensitivity $\bar{\Delta}_g$ and $\bar{\Delta}_f$ are computed.

Figure 1 shows the local partial degree of sensitivity with respect to g and f. These are related to Figs. 4A, B in the main text by

$$\Delta_g\left(y^*, f'(y^*)\right)) = 4 \cdot P\left(\text{Stable}|y^*, f'(y^*)\right) \cdot \left(1 - P\left(\text{Stable}|y^*, f'(y^*)\right)\right), \tag{8}$$

$$\Delta_f\left(g(x^*), g'(x^*)\right)) = 4 \cdot P\left(\text{Stable}|g(x^*), g'(x^*)\right) \cdot \left(1 - P\left(\text{Stable}|g(x^*), g'(x^*)\right)\right). \tag{9}$$

Computation of the conditional probabilities (and consequently the local partial degrees of sensitivity Δ_g, Δ_f) is based on considering the cross-sections V_g and V_f of the set of valid local function values V when the local function values of f and g are fixed, respectively. Since the probability distribution over V is assumed to be uniform, i.e. $\rho\left(g(x^*), g'(x^*), y^*, f'(y^*)\right) \equiv \frac{1}{\text{Volume}(V)}$, the probability of stability can be evaluated based on the proportion of the area of this cross-section which yields a stable equilibrium:

$$P\left(\text{Stable}|y^*, f'(y^*)\right) = \frac{\text{Area}\left(V_{g\,\text{Stable}}\right)}{\text{Area}\left(V_g\right)}, \tag{10}$$

$$P\left(\text{Stable}|g(x^*), g'(x^*)\right) = \frac{\text{Area}\left(V_{f\,\text{Stable}}\right)}{\text{Area}\left(V_f\right)}. \tag{11}$$

Examples of V_g are shown in Figs 2A and B. Figs 2C and D show examples of V_f.

In order to compute the partial degrees of sensitivity with respect to g and f, we need to take the expectation of Δ_g and Δ_f.

$$\bar{\Delta}_g = \int_{V_g} \rho_f\left(y^*, f'(y^*)\right) \cdot \Delta_g(y^*, f'(y^*))\, dy^* df'(y^*), \tag{12}$$

$$\bar{\Delta}_f = \int_{V_f} \rho_g\left(g(x^*), g'(x^*)\right) \cdot \Delta_f(g(x^*), g'(x^*))\, dg(x^*) dg'(x^*). \tag{13}$$

This requires the marginal probability densities

$$\rho_f\left(y^*, f'(y^*)\right) = \frac{\text{Area}(V_g)}{\text{Volume}(V)} \tag{14}$$

$$\rho_g\left(g(x^*), g'(x^*)\right) = \frac{\text{Area}(V_f)}{\text{Volume}(V)}, \tag{15}$$

which are plotted in Fig. 3A and B, respectively. The local partial sensitivities weighted by the marginal distributions, $\rho_f \cdot \Delta_g$ and $\rho_g \cdot \Delta_f$, are plotted in Fig. 4A and B, respectively.

Solving the integrals (12) and (13) gives us $\bar{\Delta}_g = 0.2148$, $\bar{\Delta}_f = 0.3260$, indicating that f is a larger source of uncertainty than g.

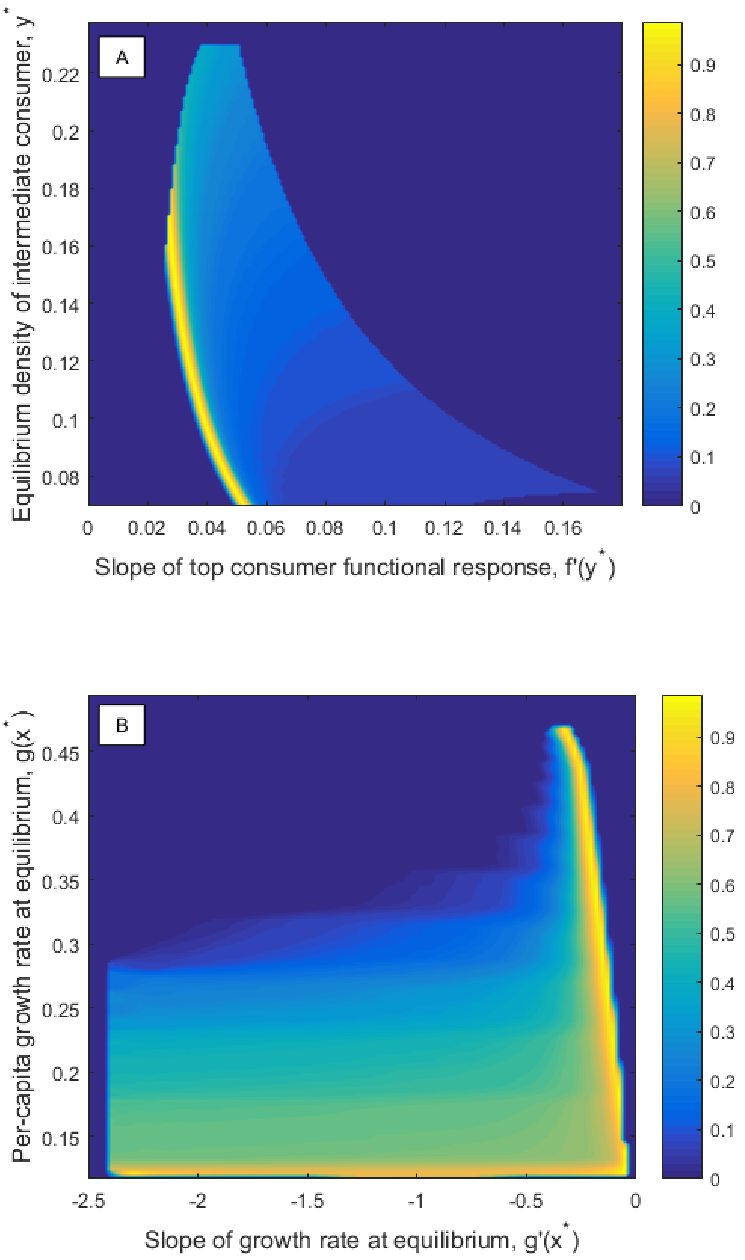

Figure 1. The local partial degree of structural sensitivity of the model (1)-(3) with respect to the two unspecified functions for maximal error bounds $\varepsilon_g^0 = 0.1$ $\varepsilon_f^0 = 0.005$. (A) With respect to variation in the per-capita growth rate of the resource g. This depends on values specified by the fixed functional response f: the equilibrium density of the intermediate consumer, y^*, and the value of f at this density, $f(y^*)$. (B) With respect to variation in the functional response of the intermediate consumer f. This depends on values specified by the fixed growth rate g: the value of g at equilibrium, $g(x^*)$, and its slope, $g'(x^*)$.

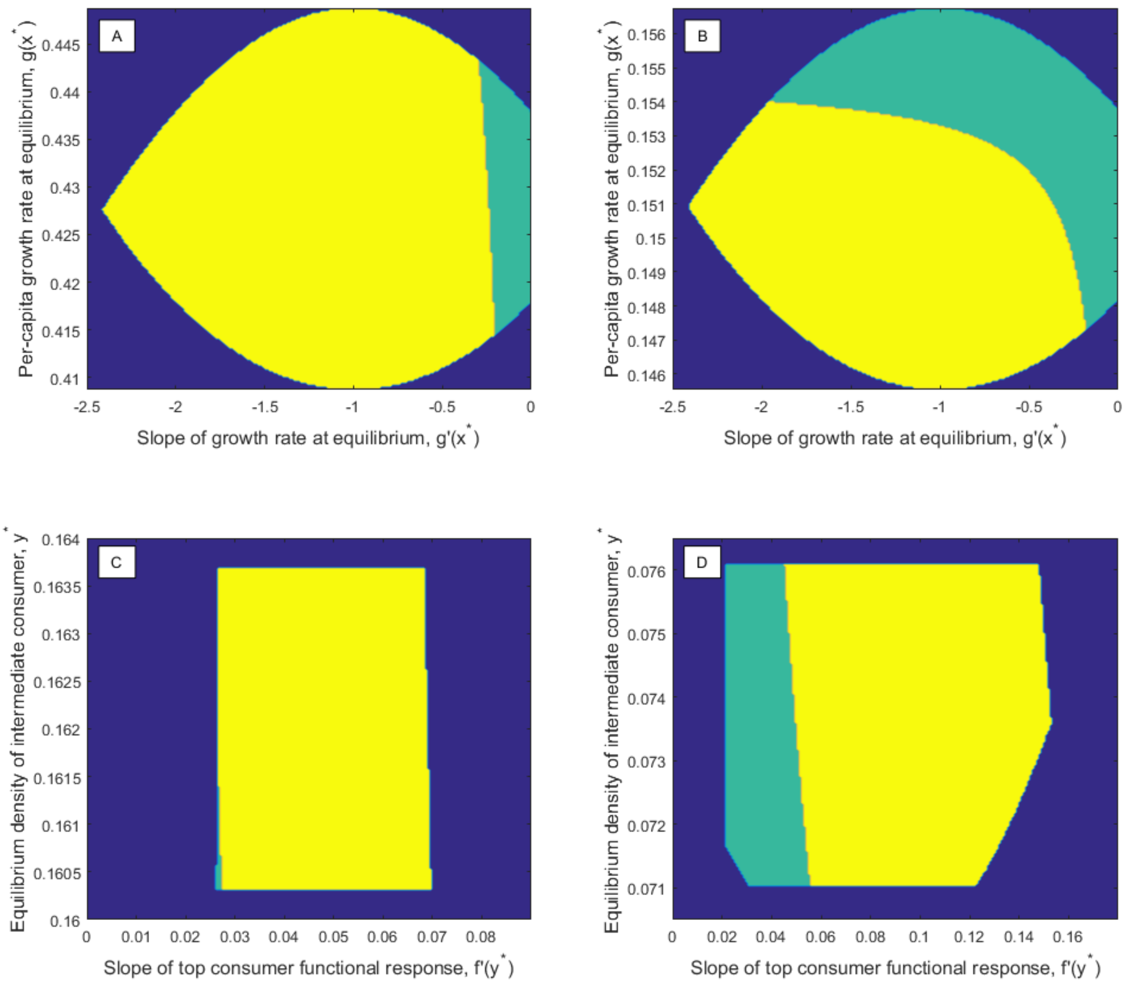

Figure 2. Cross-sections of the domain of valid functions V. Yellow and green regions denote the cross-section, with yellow indicating a stable equilibrium, and green an unstable equilibrium. All parameters are the same as in Fig. 1. Top row: Cross-sections V_g for the g-variables. (A) Low Δ_g, high ρ_f, the f-variables are fixed at $y^* = 0.22, f'(y^*) = 0.045$. (B) High Δ_g, low ρ_f, the f-variables are fixed at $y^* = 0.086, f'(y^*) = 0.045$. Bottom row: Cross-sections V_f for the f-variables. (C) Low Δ_f, low ρ_g, the g-variables are fixed at $g(x^*) = 0.3$, $g'(x^*) = -2.2$. (D) High Δ_f, high ρ_g, the g-variables are fixed at $g(x^*) = 0.1281$, $g'(x^*) = -1$.

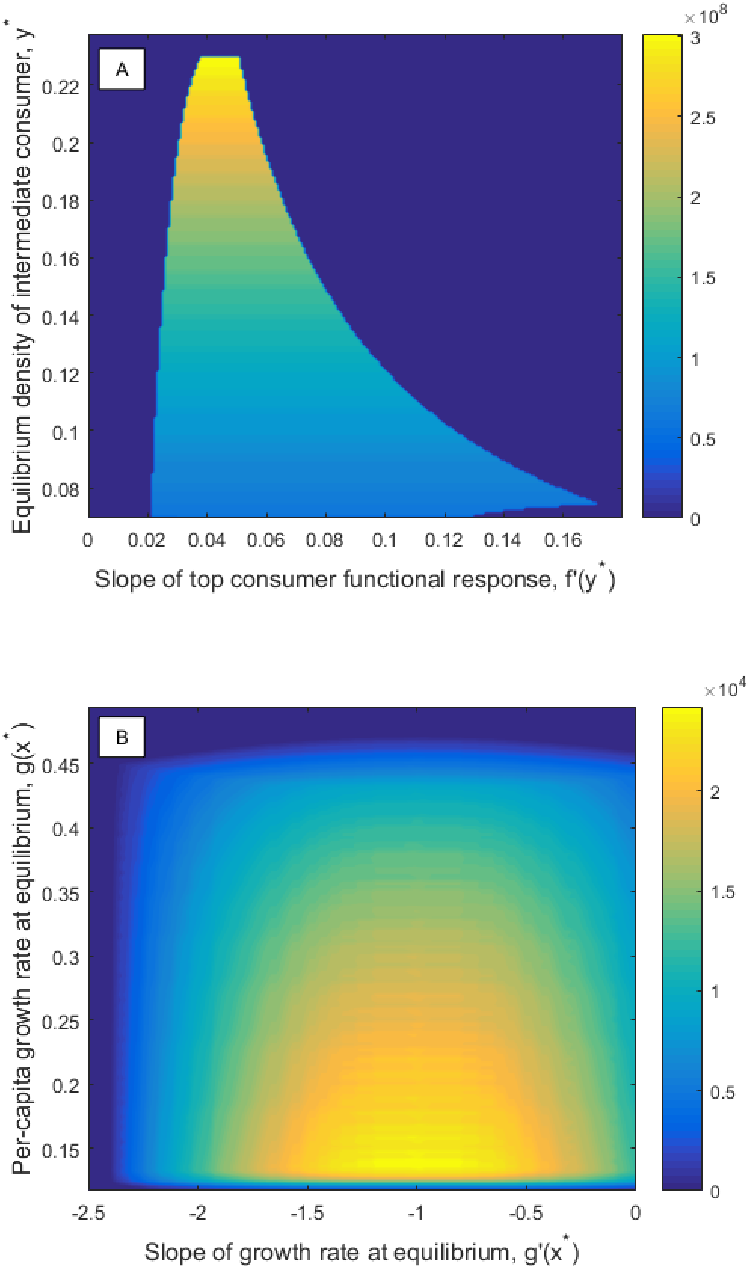

Figure 3. Marginal probability distributions of the two functions. All parameters are the same as in Fig. 1. (A) The marginal probability density of the functional response, ρ_f. ρ_f is proportional the area of V_g for the given $(y^*, f'(y^*))$. (B) The marginal probability density of the functional response, ρ_g. ρ_g is proportional the area of V_f for the given $(g(x^*), g'(x^*))$.

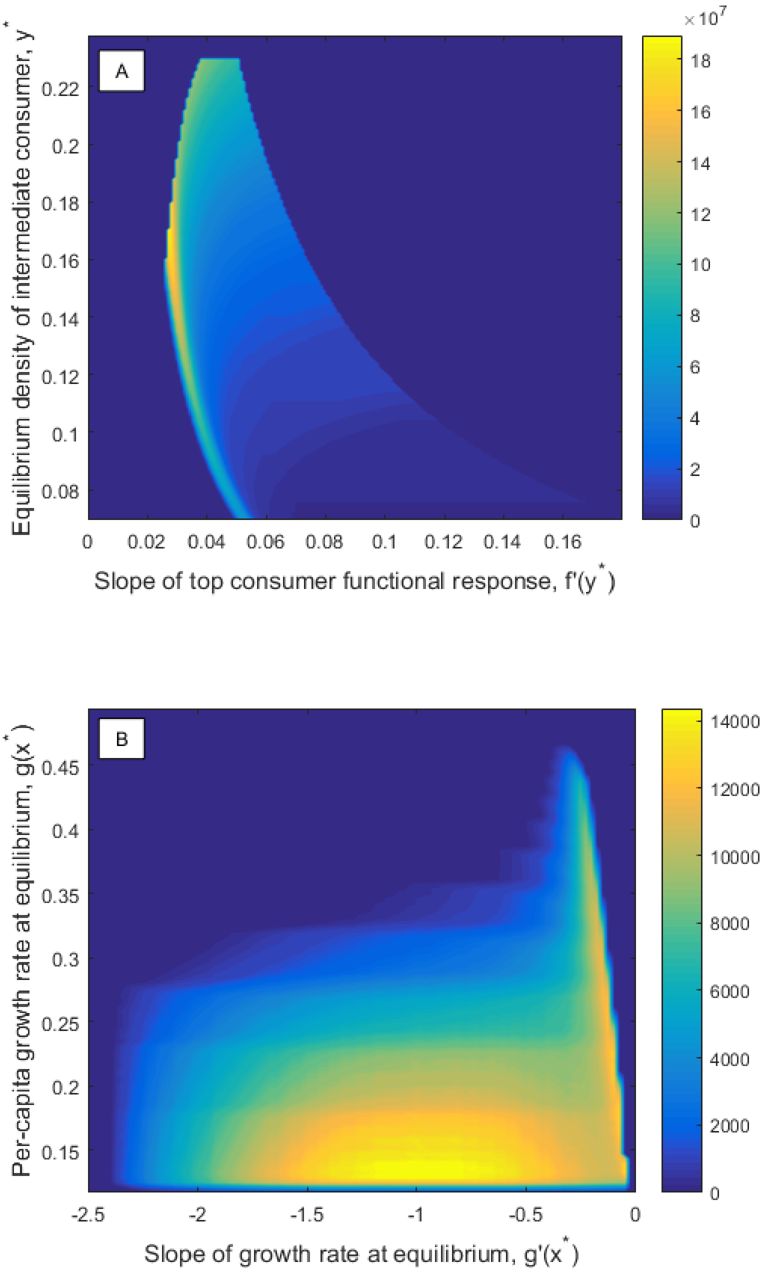

Figure 4. The local partial degrees of structural sensitivity of the model (1)-(3) weighted by the marginal distributions (A) with respect to the growth rate g, $\rho_f \cdot \Delta_g$, (B) with respect to the growth rate f, $\rho_g \cdot \Delta_f$.

C The effect of error term reduction on the partial degrees of sensitivity

In this section, we present several figures corresponding to those of the previous section, after a single iteration of the error-term reduction algorithm carried out in Section 3.3 of the main text. This gives us reduced error terms $\varepsilon_g^1 \approx 0.0835$ and $\varepsilon_f^1 \approx 0.00375$.

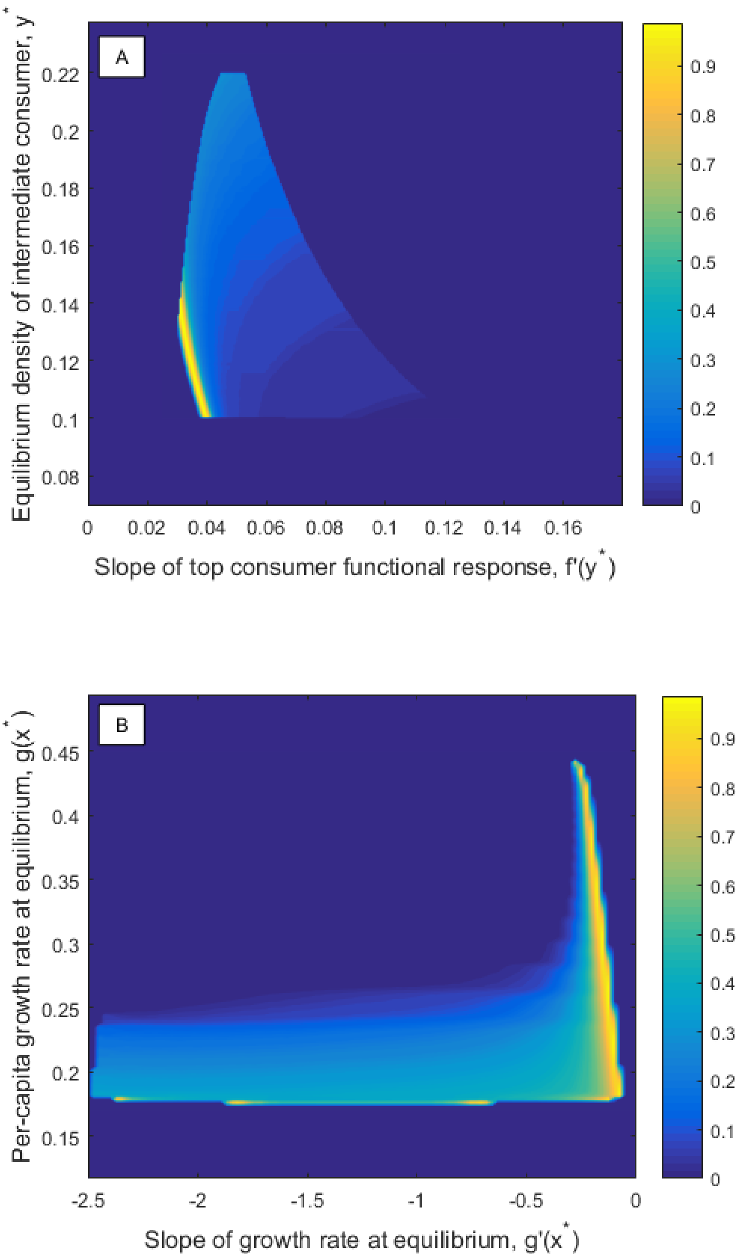

Figure 5. The local partial degree of structural sensitivity of the model (1)-(3) with respect to the two unspecified functions for reduced error bounds $\varepsilon_g^1 =\approx 0.0835$ $\varepsilon_f^1 \approx 0.00375$. (A) With respect to variation in the per-capita growth rate of the resource g, Δ_g. (B) With respect to variation in the functional response of the intermediate consumer f, Δ_f.

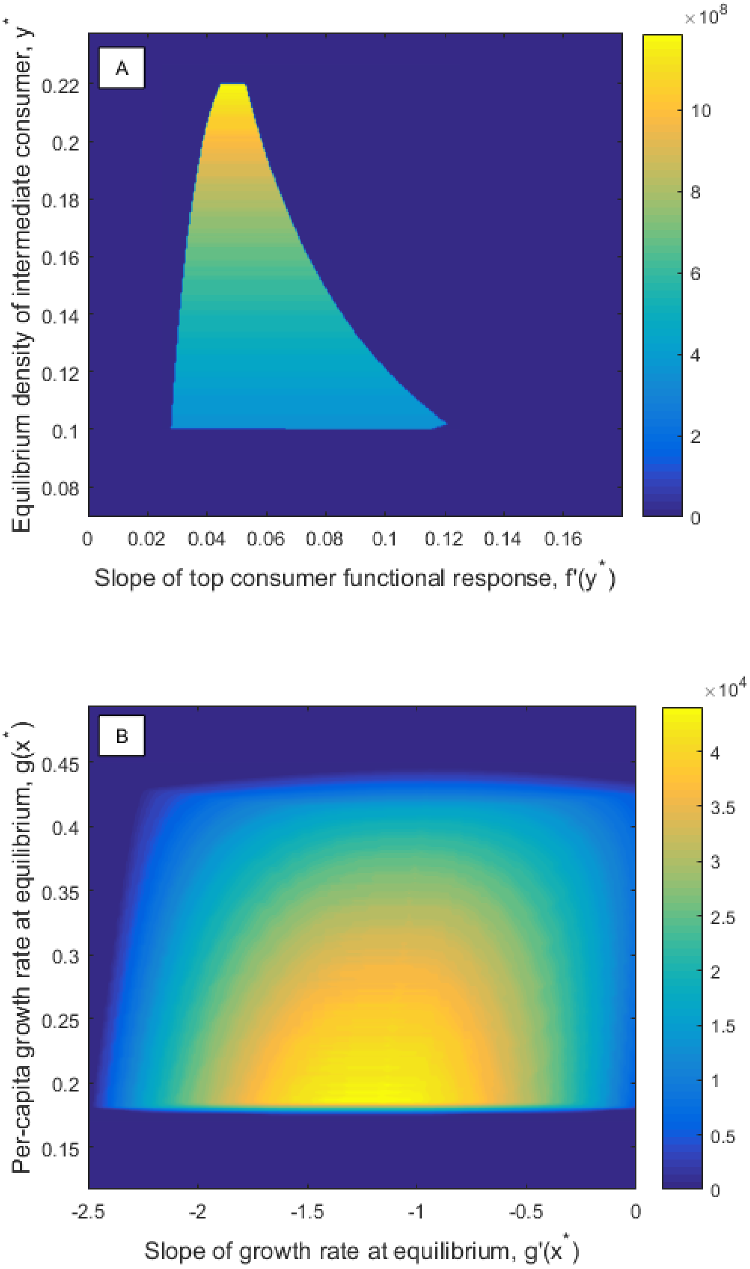

Figure 6. Marginal probability distributions of the two functions for reduced error terms $\varepsilon_g^1 \approx 0.0835$, $\varepsilon_f^1 \approx 0.00375$. All parameters are the same as in Fig. 5. (A) The marginal probability density of the functional response, ρ_f. ρ_f is proportional the area of V_g for the given $(y^*, f'(y^*))$. (B) The marginal probability density of the functional response, ρ_g. ρ_g is proportional the area of V_f for the given $(g(x^*), g'(x^*))$.

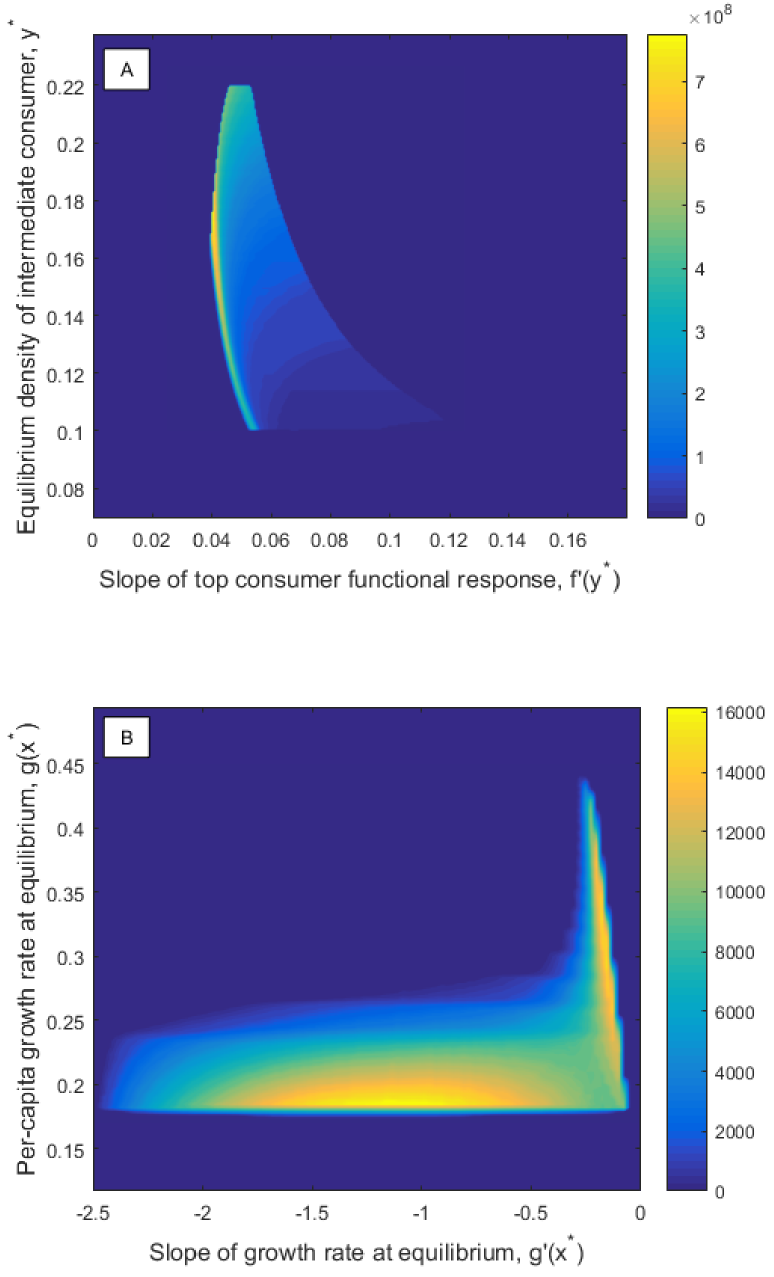

Figure 7. The local partial degrees of structural sensitivity of the model (1)-(3) weighted by the marginal distributions, for reduced error terms $\varepsilon_g^1 \approx 0.0835$, $\varepsilon_f^1 \approx 0.00375$ (A) with respect to the growth rate g, $\rho_f \cdot \Delta_g$, (B) with respect to the growth rate f, $\rho_g \cdot \Delta_f$.

References

1. Hastings, A. & Powell, T. Chaos in a Three-Species Food Chain. *Ecology* **72**, 896–903, DOI: 10.2307/1940591 (1991).
